# Benign tumors in TSC are amenable to treatment by GD3 CAR T cells in mice

**DOI:** 10.1172/jci.insight.152014

**Published:** 2021-11-22

**Authors:** Ancy Thomas, Saurav Sumughan, Emilia R. Dellacecca, Rohan S. Shivde, Nicola Lancki, Zhussipbek Mukhatayev, Cristina C. Vaca, Fei Han, Levi Barse, Steven W. Henning, Jesus Zamora-Pineda, Suhail Akhtar, Nikhilesh Gupta, Jasmine O. Zahid, Stephanie R. Zack, Prathyaya Ramesh, Dinesh Jaishankar, Agnes S.Y. Lo, Joel Moss, Maria M. Picken, Thomas N. Darling, Denise M. Scholtens, Daniel F. Dilling, Richard P. Junghans, I. Caroline Le Poole

**Affiliations:** 1 Department of Dermatology, Feinberg School of Medicine;; 2 Robert H. Lurie Comprehensive Cancer Center;; 3 Quantitative Data Sciences Core, Robert H. Lurie Comprehensive Cancer Center; and; 4 Department of Pharmacology, Feinberg School of Medicine, Northwestern University, Chicago, Illinois, USA.; 5 Department of Microbiology and Immunology, Stritch School of Medicine, Loyola University, Maywood, Illinois, USA.; 6 Illinois Mathematics and Science Academy, Aurora, Illinois, USA.; 7 Department of Medicine, Beth Israel Deaconess Medical Center, Harvard Medical School, Boston, Massachusetts, USA.; 8 Pulmonary Branch, National Heart, Lung, and Blood Institute, NIH, Bethesda, Maryland, USA.; 9 Department of Pathology, Loyola University, Maywood, Illinois, USA.; 10 Department of Dermatology, School of Medicine, Uniformed Services University, Bethesda, Maryland, USA.; 11 Department of Preventive Medicine, Feinberg School of Medicine, Northwestern University, Chicago, Illinois, USA.; 12 Department of Medicine, Stritch School of Medicine, Loyola University, Maywood, Illinois, USA.; 13 Department of Hematology/Oncology, School of Medicine, Boston University, Boston, Massachusetts, USA.; 14 Department of Microbiology-Immunology, Feinberg School of Medicine, Northwestern University, Chicago, Illinois, USA.

**Keywords:** Immunology, Therapeutics, Antigen, Immunotherapy, T cells

## Abstract

Mutations underlying disease in tuberous sclerosis complex (TSC) give rise to tumors with biallelic mutations in *TSC1* or *TSC2* and hyperactive mammalian target of rapamycin complex 1 (mTORC1). Benign tumors might exhibit de novo expression of immunogens, targetable by immunotherapy. As tumors may rely on ganglioside D3 (GD3) expression for mTORC1 activation and growth, we compared GD3 expression in tissues from patients with TSC and controls. GD3 was overexpressed in affected tissues from patients with TSC and also in aging *Tsc2+/*^–^ mice. As GD3 overexpression was not accompanied by marked natural immune responses to the target molecule, we performed preclinical studies with GD3 chimeric antigen receptor (CAR) T cells. Polyfunctional CAR T cells were cytotoxic toward GD3-overexpressing targets. In mice challenged with *Tsc2*^–/–^ tumor cells, CAR T cells substantially and durably reduced the tumor burden, correlating with increased T cell infiltration. We also treated aged *Tsc2+/*^–^ heterozygous (>60 weeks) mice that carry spontaneous *Tsc2*^–/–^ tumors with GD3 CAR or untransduced T cells and evaluated them at endpoint. Following CAR T cell treatment, the majority of mice were tumor free while all control animals carried tumors. The outcomes demonstrate a strong treatment effect and suggest that targeting GD3 can be successful in TSC.

## Graphical abstract

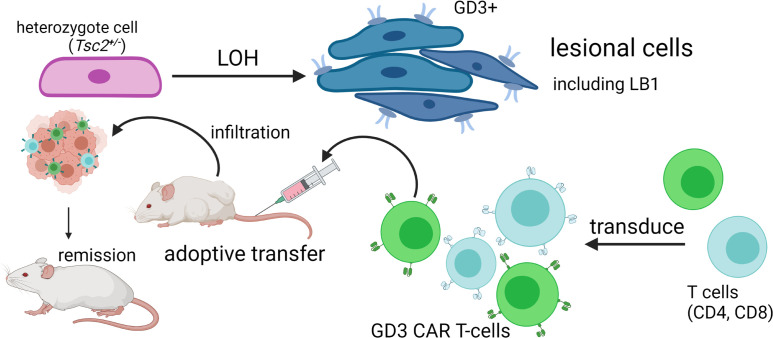

## Introduction

Tuberous sclerosis complex (TSC) is an autosomal dominant, multisystem genetic disorder. TSC affects children and adults, with an estimated incidence at birth of approximately 1 in 6000 and a prevalence of 1 in 14,000 (1), affecting about 1.5 million individuals worldwide. TSC and lymphangioleiomyomatosis (LAM) are caused by biallelic mutations of the *TSC1* or *TSC2* genes, coding for tumor suppressors hamartin and tuberin, respectively. Upon loss of heterozygosity, biallelic mutations in a single gene give rise to benign tumors in vital organs, associated with constitutive activation of mammalian target of rapamycin complex 1 (mTORC1) (1). Clinical manifestations of TSC vary from skin discolorations to progressive development of tumors in multiple organs, including lungs, as in TSC-associated and sporadic LAM (2). Neurological symptoms include epilepsy and long-term memory impairment (3), TSC-associated neuropsychiatric disorders (4), and autism (5). The tumors are generally benign, growing slowly compared with full-blown cancer. However, tumors develop in any location, causing significant morbidity (6). TSC further predisposes patients to more aggressive tumors, including renal cell carcinoma (7). Thus, there is an unmet need for effective treatment modalities (8).

There is currently no cure for TSC, though some treatments can combat symptoms. Rapamycin and derivative mTOR inhibitors have been deployed for patients with TSC. Rapalogs stabilize lung function, reduce skin lesions, and decrease the size of renal angiomyolipomas and lymphatic lesions (9). However, rapalogs are not cytotoxic to tumor cells. Moreover, resulting mTORC2 inhibition is linked to insulin resistance and a risk of type 2 diabetes (10). Treatment is also associated with increased risk of infections (11). These effects can ultimately reduce the quality of life and preclude patients from long-term treatment. Treatment withdrawal causes tumor regrowth in most patients (12).

TSC is a monogenic disease (13), and tumor cells with biallelic *TSC1* or *TSC2* mutations generally do not develop further heritable changes in other genes. Compared with hypermutated malignancies, TSC-associated tumors exhibit a predictable phenotype (14). A gene product expressed specifically by cells with *TSC* mutations may be targetable by immunotherapy. A critical step toward applying immunotherapy to benign tumors will thus be to identify target molecule(s) recognizable by the adaptive immune system, leaving healthy tissues intact.

Ganglioside D3 (GD3) is a membrane glycosphingolipid expressed during normal brain development and in several malignancies (15). We observed overexpression of GD3 in LAM, wherein mutations in *TSC1* or *TSC2* are responsible for tumor development (16). In LAM, however, multiple options for antigen-specific immune targeting exist as pulmonary lesions express melanogenic enzymes that are immunogenic in vitiligo and melanoma (17, 18). In TSC, the additional challenge remains to identify a target molecule expressed by lesions in every location, while engaging a therapeutic with the potential to overcome immune privilege (19).

GD3 expression accelerates tumor growth (20). We propose that GD3 overexpression develops in response to platelet-derived growth factor (PDGF) stimulation of *TSC2*^–/–^ cells and provides a survival advantage to host cells. PDGF is upregulated in TSC2-deficient cells (21). In the presence of reactive oxygen species, PDGF will induce IL-13 expression (22), which stimulates GD3 expression (23). GD3 colocalizes with PDGF receptor (PDGFR), forming a complex enhancing PDGF signals to promote proliferation and invasion (24). This could particularly influence tumorigenesis of TSC2-deficient cells, wherein PDGFR expression is reduced (25).

Immunotherapy has been used to treat hematologic malignancies. The US Food and Drug Administration has approved chimeric antigen receptor (CAR) T cell therapy for hematologic cancers expressing CD19 (15). The success of adoptively transferred, genetically modified T cells in hematologic malignancies has raised hopes that durable remissions may be achieved for patients with solid tumors, including tumors in TSC and LAM. The effects of treatment can be long-lived, because CAR T cell treatment employs reproducing, live cells capable of memory formation (16). In this context, a molecule overexpressed by TSC tumor cells can be an attractive target for immunotherapy.

As mTORC1 hyperactivity is likely associated with enhanced GD3 expression, we investigated GD3 abundance in TSC-affected tissues. As a lipid antigen, GD3 can be processed and presented to NKT cells by CD1d, expressed on antigen-presenting cells. Promoting NKT cell activity may help keep GD3-expressing tumors in check (26). Thus, we probed NKT cell abundance, as well as T cell and NK cell infiltration to TSC-affected tissues, to understand whether the tumors are “hot,” or could benefit from passive immunotherapy. In this respect, monoclonal antibodies against cell surface gangliosides have been tested as therapeutics (27). Moreover, second- and third-generation CAR T cells that recognize GD3 have shown efficacy in preclinical melanoma models (28). We questioned whether CAR T cells might benefit the treatment of benign tumors in a mouse model of TSC. This bears special significance given the impact of altered mTOR signaling on T cell function and memory formation (29). However, monoallelic mutations appear less impactful, and T cell functions seem largely preserved in patients overall (30).

We obtained human pulmonary, renal, brain, and cutaneous tissues from patients with TSC to evaluate GD3 expression compared with healthy control tissues. Then, we measured the abundance of NKT cells and other immune cells in control and TSC-affected tissues, evaluated CD1d expression, and estimated existing serum antibody titers against GD3 in patients and controls. Next, we engineered T cells to express a second-generation GD3 CAR molecule. When applied to a mouse model of melanoma, GD3 CAR T cells operated without side effects (28). Here, GD3 CAR T cells were administrated by adoptive transfer to target TSC- associated tumors in immunodeficient mice. CAR T cells were also employed to treat spontaneously arising tumors that occur due to loss of heterozygosity in aging *Tsc2+/*^–^ mice. Tumor infiltration by (transgenic) T cells was evaluated and aligned with remaining GD3 expression.

The data can serve to establish the treatment potential of GD3 CAR T cells as a viable option for tumor control in TSC. More broadly, these experiments serve to test the concept that benign tumors might be amenable to immunotherapy.

## Results

### GD3 is consistently overexpressed in diseased TSC tissue.

**Figure 1:**
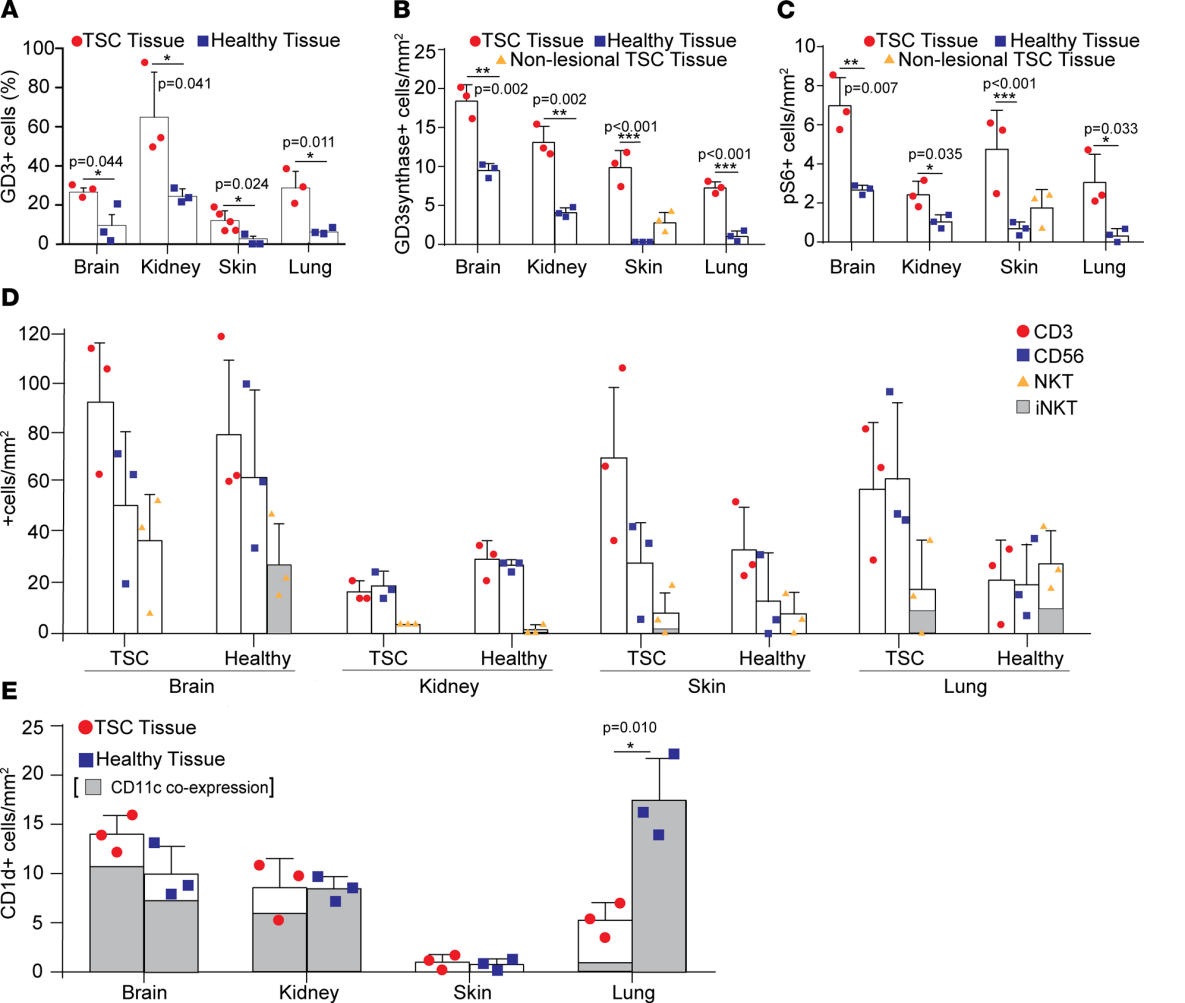
GD3 is overexpressed in human TSC lesions, and GD3 overexpression fails to raise cellular immune responses to the antigen in human TSC tissues. (**A**) Percentage of GD3-expressing cells quantified (%). (**B**) GD3 expression quantified as GD3 synthase–positive cells per area. (**C**) Quantification of TSC lesional cells as phospho-S6^+^ cells per area. (**D**) Quantification of T cells and NK cells shown as CD3^+^ and CD56^+^; NKT cells are evaluated as CD3/CD56 double-positive cells in kidney tissue (enlarged representation) with iNKT cells quantified as CD3^+^ and CD56^+^, TCR Vα24-Jα18^+^ cells. (**E**) Quantification of CD1d- and CD1d-CD11c–expressing cells per area. Statistical analysis was performed using 2-tailed Student’s *t* tests assuming equal variance among groups. Where 3 tissue sources were displayed, we performed a 1-way ANOVA (***P < 0.001) followed by Holm-Šídák multiple comparisons test to compare TSC-affected tissues with controls. In all quantifications, *n* = 3; *P < 0.05, **P < 0.01, ***P < 0.001. Graphs show individual values and mean values ± SD.

Previous reports demonstrated that expression of GD3 was observed in tumors, including those observed in LAM (16). Due to the genetic link between TSC and LAM and our previous observations of GD3 expression in *Tsc2*^+/–^ heterozygote mice (16), we hypothesized that GD3 may also be an immunotherapeutic target for patients with TSC. To test this, we first examined GD3 expression in affected tissues from patients with TSC and healthy tissues from control donors, 3 tissues from each group. As shown in Figure 1A and Supplemental Figure 1A (supplemental material available online with this article; https://doi.org/10.1172/jci.insight.152014DS1), among brain, kidney, skin, and lung tissues, the TSC tumor tissues held a significantly greater number of GD3-expressing cells per square millimeter than healthy control tissues. Consistent with GD3 expression, GD3 synthase, the enzyme responsible for GD3 synthesis from GM3, was also overexpressed in brain, kidney, skin, and lung tissues (Figure 1B).

Since TSC lesions exhibit hyperactivation of mTORC1, we also assessed phospho-S6 expression as a downstream indicator of increased mTORC1 activity to locate the lesions (31). We found that tissues which expressed higher GD3 also had increased phospho-S6 expression, consistent with disease activity in TSC (32) (Figure 1C). Moreover, we also observed colocalization of phospho-S6 and GD3 in tissues obtained from patients diagnosed with TSC (Supplemental Figure 1B), suggesting that GD3 expression is associated with mTORC1 activation in TSC lesional cells (33).

Taken together, this analysis demonstrates a greater abundance of GD3 expression in TSC tumor tissues, suggesting the plausibility of a GD3-targeted treatment of TSC.

### TSC does not cause significant changes in NKT cell infiltration.

Since GD3 can be cross-presented by antigen-presenting cells (APCs) and induce iNKT cell responses in a melanoma mouse model (34), we examined NKT cell infiltration in the microenvironment of TSC patient tissues. We also quantified (CD3 expressing) T cells and (CD56 expressing) NK cells while examining (CD3/CD56 double positive) NKT cell and invariant TCR coexpression in tissues from both healthy donors and patients with TSC (*n* = 3). We found similar numbers of CD3^+^, CD56^+^, and CD3/CD56 double-positive cells in brain, kidney, skin, and lung tissues from patients with TSC compared with control tissues (Figure 1D), composed of similar percentages of iNKT cells as determined by coexpression of the invariant TCR as shown in Supplemental Figure 1C.

Despite marked overexpression of GD3 in TSC-derived tissues, the absence of TSC expression does not lead to a significant increase in immune responses to tumor cells. This lack of evidence for a natural immune response to GD3 leads us to believe that introducing an immune response to the ganglioside can help overcome this deficiency to effectively treat TSC and eliminate tumor tissue.

### Limited NKT infiltration is not due to a lack of CD1d expression in TSC tissues.

As a glycolipid antigen, GD3 can be processed and presented by CD1d-expressing APCs and might recruit responding NKT cells. To understand whether tumors in TSC experience infiltration by relevant immune cells, we evaluated the presence and abundance of CD1d-expressing cells, including CD11c^+^ APCs (*n* = 3). The CD1d^+^ APCs were present in both normal and TSC brain, kidney, skin, and lung tissues, although their abundance was not significantly changed compared with healthy tissues, except in the lung (Figure 1E and Supplemental Figure 1D).

The data suggest that CD1d^+^ APCs are present in TSC tissues and may present GD3 to NKT cells but are not prompted to do so by overexpression of GD3. Nevertheless, TSC lesions may be targeted by NKT cells in response to vaccination as we have proposed previously (26).

### Patients and mice with TSC do not exhibit enhanced anti-GD3 serum titers.

**Figure 2:**
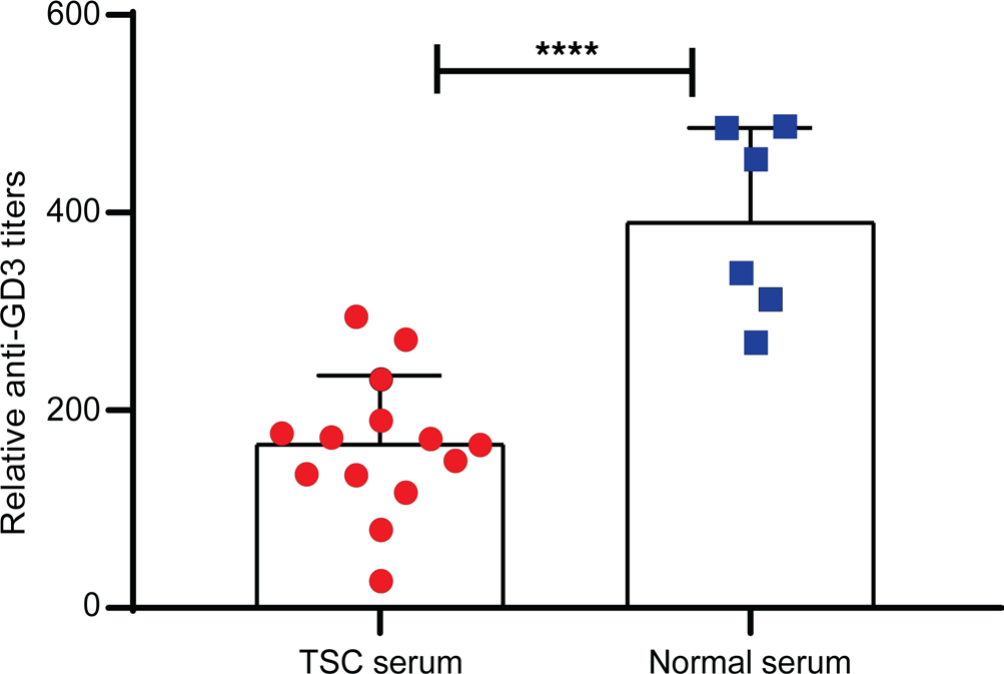
TSC patients exhibit reduced anti-GD3 titers compared with controls. Antibody titers were quantified in serum samples from 14 patients with TSC and compared with 6 healthy controls as measured by ELISA and compared by a 2-tailed Student’s *t* test assuming equal variance, *n* = 14. ****P < 0.0001.

Circulating GD3 antibody titers were examined in serum samples from 14 patients with TSC and 6 healthy donors. We found lower GD3 antibody titers in patients with TSC compared with control sera (Figure 2). The same was previously observed in sera from patients with melanoma or LAM (16). Because such antibodies could otherwise bind GD3-expressing target cells to mediate complement-mediated cytotoxicity (23) or induce antibody-dependent cellular cytotoxicity, reduced titers in patients reflect a lack of humoral responses to the GD3 molecule and might offer an opportunity for TSC tumor cells to escape immune surveillance. Aging *Tsc2*^+/–^ mice did not exhibit elevated antibody titers against GD3 (Supplemental Figure 2). This prompted us to investigate opportunities for cellular therapies to keep tumor growth in check.

### GD3 CAR T cells are cytotoxic toward GD3-expressing target cells in vitro.

**Figure 3:**
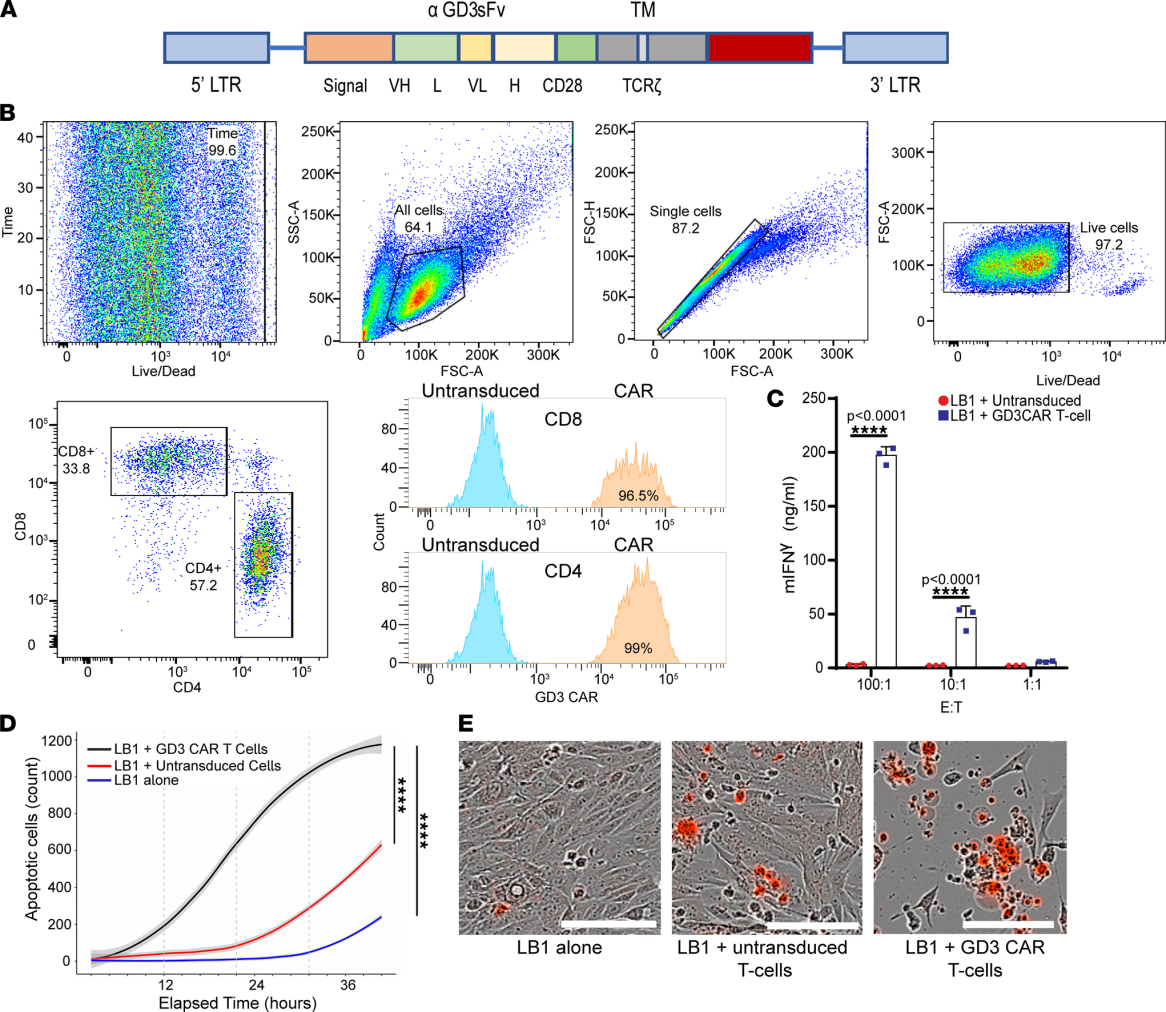
GD3 CAR T cells respond to antigen in vitro. (**A**) Schematic representation of the GD3 CAR construct used. Fv, variable domain of antibody; TM, transmembrane domain; LTR, long terminal repeat. (**B**) Efficient transduction of both CD4^+^ and CD8^+^ T cells by our second-generation GD3 CAR. (**C**) IFN-γ secretion by GD3 CAR T cells in response to LB1 *Tsc2*^–*/*–^ mouse kidney tumor cells. This experiment was performed 3 times with similar results. A representative experiment is shown. Results were analyzed by 2-way ANOVA followed by Bonferroni’s multiple comparisons test. m, murine; E:T, effector/target ratio. (**D**) Cytotoxicity of GD3 CAR T cells toward LB1 *Tsc2*^–/–^ mouse kidney tumor cells when cocultured at an E:T ratio of 10:1. A generalized linear mixed model with log link and 0-inflated quasi-Poisson distribution assumption was used and included fixed effects for treatment group and a random effect for wells. Three images/well were acquired every 3 hours for 48 hours, and apoptotic cells stained by caspase-7 red dye were quantified. ****P < 0.0001. (**E**) Representative images of target cell death (red) induced by GD3 CAR T cells and untransduced T cells, scale bar: 400 μm.

Since TSC lesions overexpress cell surface antigen GD3, we pursued targeting the ganglioside by CAR T cells as a new approach for TSC treatment. To test our hypothesis, we transduced primary mouse T cells with a retroviral construct (Figure 3A) encoding a second-generation GD3-reactive CAR (26) and monitored the reactivity of GD3 CAR T cells to relevant target cells expressing GD3 in vitro. CAR transduction efficiency in primary mouse T cells was evaluated by flow cytometry using a polyclonal antibody against the unique sequence involved in binding of the CAR to GD3 (35).

As a representative transduction result, the cells represented in Figure 3 rendered a 99% transduction efficiency of CD4^+^ T cells and 96.5% of CD8^+^ T cells (Figure 3B). In contrast, the untransduced T cell group showed negligible GD3 CAR background expression. Transduced mouse T cells were efficiently expanded in vitro and proliferated at similar rates as untransduced mouse T cells (Supplemental Figure 3, A–C), amplifying 9.3-fold over 10 days compared with 7.4-fold for untransduced T cells.

The production of mouse IFN-γ by the GD3 CAR T cells was measured upon activation with various target cells, including GD3-overexpressing LB1*Tsc2^–/–^* tumor cells. Consistent with the GD3 CAR T cell–mediated cytotoxicity, IFN-γ production was increased from 0 to 200 ng/mL compared with polyclonal effectors at 100:1 E:T ratios (Figure 3C). Tukey’s multiple comparisons test identified CAR T cells added at a 100:1 ratio as secreting significantly more IFN-γ than untransduced T cells while the difference to CAR T cells added at a 10:1 ratio was also significant. To further evaluate the targeting efficacy of GD3 CAR T cells, we also used HEK293 cells overexpressing GD3 synthase by transfection (26). We cocultured different target cell lines with untransduced and GD3 CAR T cells at several E:T ratios. The assessment of cytokine secretion revealed greatly enhanced IFN-γ secretion by GD3 CAR T cells compared with untransduced T cells (Supplemental Figure 4A). Comparing apoptosis over 48 hours indicated significantly increased apoptosis when HEK293 GD3 synthase cells were cocultured with GD3 CAR T cells compared with untransduced T cells (Supplemental Figure 4, B and C). Greater significance was observed when *Tsc2^–/–^* tumor cells were used as targets (Figure 3, D and E). A similar effect was observed with GD3-expressing human melanoma cells as control targets (Supplemental Figure 5).

Both CD8^+^ and CD4^+^ T cells were efficiently transduced to express the GD3 CAR, and we next evaluated whether both CAR T cell subsets differentially responded to cognate antigen-expressing target cells, as a contribution for CD4^+^ CAR T cells to antitumor responses might be expected (36).

### Polyfunctional responses are observed in both CD4^+^ and CD8^+^ CAR T cells responding to antigen.

**Figure 4:**
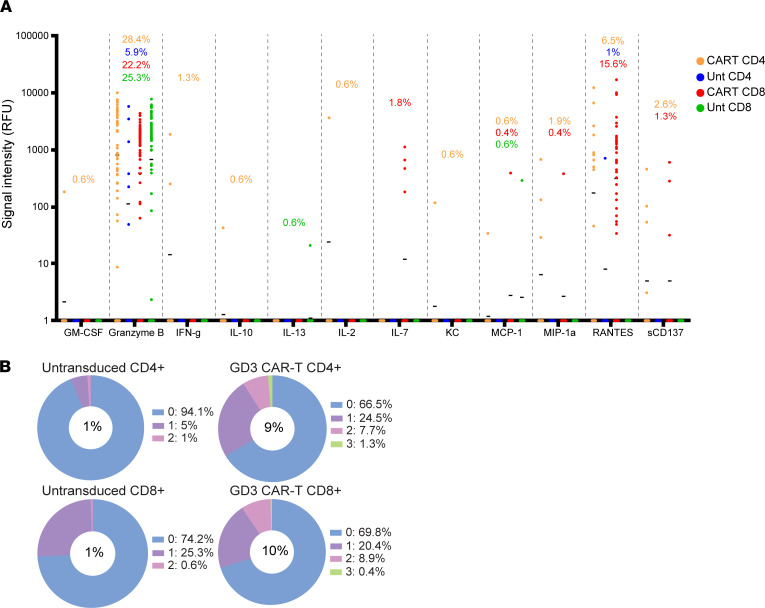
Polyfunctional responses in CD4^+^ and CD8^+^ CAR T cells in response to antigen. CD4 and CD8 subsets of untransduced or GD3 CAR transduced T cells were magnetically separated and individually cocultured 2:1 with GD3-expressing HEK293 cells for 20 hours before loading onto IsoCode chips. (**A**) Wells containing single live cells were analyzed by IsoSpeak data visualization software, and detection of individual analytes is represented by signal intensity per cell. Horizontal bars denote the mean for each analyte. (**B**) The percentages of single T cells secreting the indicated number of cytokines are shown in pie charts, with the percentage of polyfunctional T cells (expressing 2 or more cytokines) listed in the center of the pie.

Polyfunctional T cells express multiple (2 or more) cytokines, and their presence is associated with functions including effective tumor control and antitumor responses (37, 38). The immune functional properties of the untransduced and GD3 CAR T cells were compared after antigen stimulation by GD3-expressing target cells. T cells were separated from their targets before polyfunctionality among CD4^+^ and CD8^+^ T cell subsets was measured using IsoCode mouse chips to probe the secretion of up to 28 cytokines at the single–T cell level. Overall, transduced T cells expressed a broader cytokine repertoire than untransduced T cells of either subtype (Figure 4A). Moreover, among both CD4^+^ and CD8^+^ transduced T cell subsets, enhanced polyfunctionality was detected when compared with the untransduced T cells of the same subpopulation (Figure 4B).

Defining the polyfunctional strength index (PSI) as the percentage of cells expressing 2 or more cytokines multiplied by the strength of the signal (measured as mean fluorescence intensity, or MFI) for each cytokine, the PSI value was increased among T cells of both the CD4 and CD8 subsets. For untransduced and CAR T cells, the PSI was 15 and 133, respectively, among CD4^+^ T cells. Meanwhile CD8^+^ T cells held a PSI of 7 for untransduced T cells and 150 for CAR T cells. In the CD4^+^ population, the percentage of cells expressing 2 cytokines increased from 1% to 7.7%, and 1.3% of the transduced cells secreted 3 cytokines compared with none among untransduced T cells. Similarly, 8.9% of the transduced CD8^+^ CAR T cells secreted 2 cytokines, and 0.4% secreted 3 cytokines per cell whereas only 0.6% of the untransduced T cells generated 2 cytokines. Thus, the polyfunctionality was increased 9- and 10-fold among GD3 CAR T cells in the CD4 and CD8 populations, respectively. This was mainly attributed to effector, chemoattractive, and stimulatory cytokines in both CD4 and CD8 subsets of the GD3 CAR T cells, with an appreciable enhancement of granzyme B and RANTES secretion. Supplemental Figure 6 provides additional granularity. Separating the untransduced and transduced populations among CD4^+^ and CD8^+^ T cells, the t-distributed stochastic neighbor embedding plots show the cytokine expression profiles and production levels for individual cytokines. For the CD4 population, enhanced secretion of RANTES and granzyme B among transduced T cells was the most prominent observation. Among CD8^+^ T cells, the cytokine profiles showed a greater shift toward de novo and increased secretion of granzyme B, IFN-γ, IL-7, macrophage inflammatory protein 1α, and RANTES secretion on a per-cell basis. In sum, both CD4^+^ and CD8^+^ CAR T cells can respond to GD3 in vitro, and their combined effects might contribute to antitumor responses in models of TSC.

### GD3 CAR T cells inhibit Tsc2^–/–^ tumor growth in immunodeficient mice.

**Figure 5:**
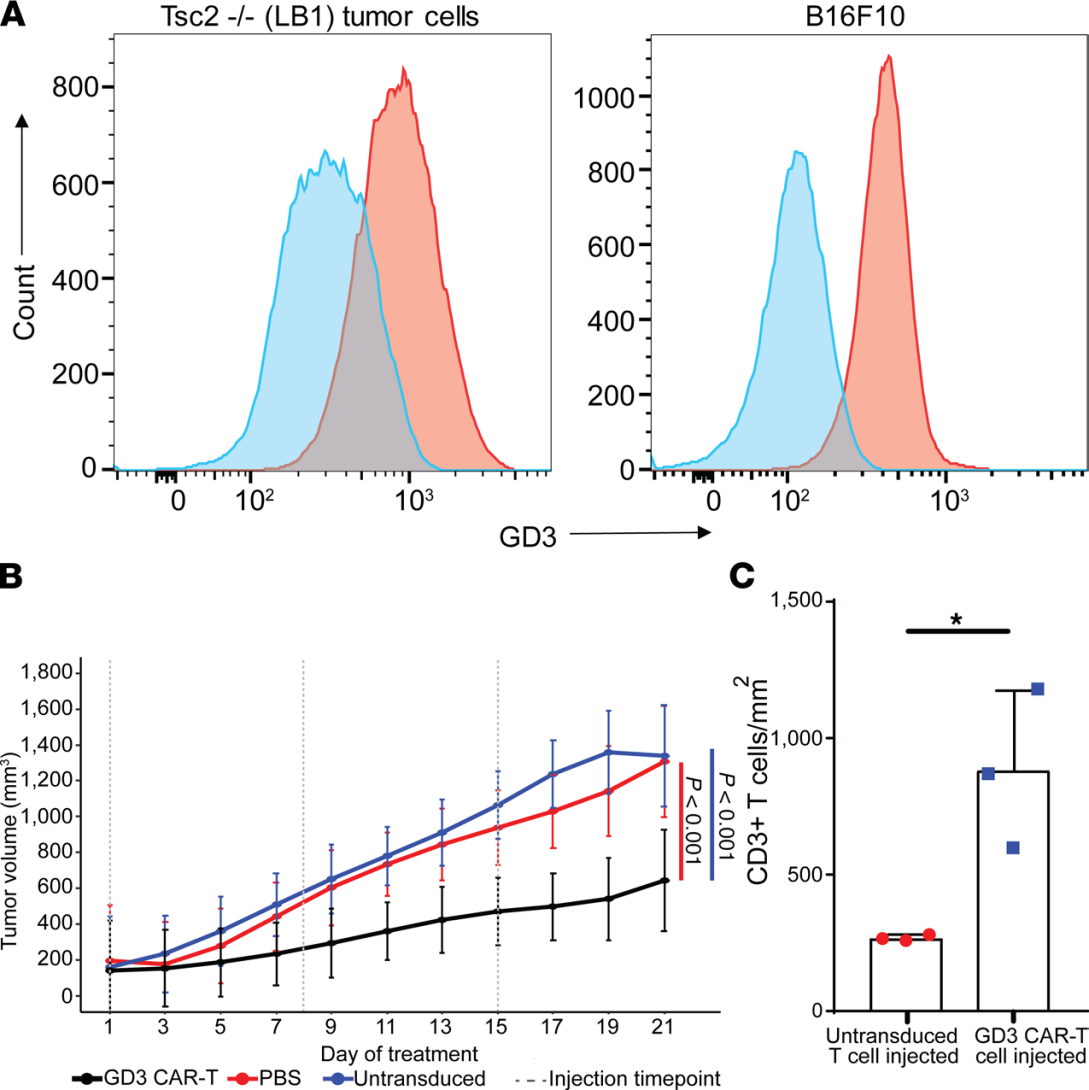
GD3 CAR T cells target *Tsc2^–/–^* tumors in immunodeficient SCID/Bg mice. (**A**) Surface GD3 expression by LB1 *Tsc2*^–*/*–^ kidney tumor cells used for subcutaneous tumor injections, and B16-F10 mouse melanoma cells for comparison with a shift of 545 in MFI for LB1 and a shift of 298 for B16-F10, representing a 2.7- and 3.6-fold increase in fluorescence over background for LB1 cells and B16 cells, respectively. (**B**) Tumor volumes in the treatment groups over time. Figure shows data from a representative experiment of 2 performed. Each group had 6 animals, and tumor volumes were documented every other day for 22 days after the first T cell treatment. Statistical analysis was carried out using R software, and splines were fit using the splines2 package. Two-sided *P* values are provided using a Bonferroni correction, with *P* values of *P* = 3.3 × 10^–6^ for the comparison of CAR T cells with PBS and *P* = 3.2 × 10^–15^ for the comparison of GD3 CAR T cells with the untransduced T cell group. (**C**) Quantification of CD3^+^ T cells infiltrating subcutaneous tumors after adoptive T cell transfer in SCID/Bg mice that received untransduced or GD3 CAR T cells, *n* = 3. *P = 0.022. A 2-tailed Student’s *t* test assuming equal variance was used to compare groups. Bar graphs show mean values ± SD.

To evaluate the efficacy of CAR T cells in vivo, a xenograft model from an immunodeficient mouse challenged with *Tsc2^–/–^* tumor cells was used. SCID/Beige mice received 1 × 10^6^ LB1 *Tsc2^–/–^* tumor cells expressing GD3 (Figure 5A) subcutaneously, and tumor development was visible in 10–15 days (~6 mm^3^). Then, tumors were measured every other day, and the mice were randomly distributed among treatment groups to receive GD3 CAR T cells, untransduced T cells, or PBS (*n* = 6). The tumor volume plotted over time indicated a significant treatment effect of GD3 CAR T cells compared with either control group (PBS and untransduced T cell recipients) (Figure 5B). Tumor volumes in individual mice are represented in Supplemental Figure 7.

Significantly increased T cell infiltration was found in tumors of GD3 CAR T cell–treated mice compared with untransduced T cell recipients (*n* = 3) (Figure 5C and Supplemental Figure 8A). PBS-treated animals showed no T cell infiltration to the tumor. Supplemental Figure 8B shows a representative image that confirms the expression of GD3 in a tumor from the control (PBS-treated) mice. Tumor-infiltrating GD3 CAR T cells were readily detected in the CAR T–injected mice (Supplemental Figure 8C).

### GD3 CAR T cells limit spontaneous tumor in aging Tsc2^+/–^ mice.

**Figure 6:**
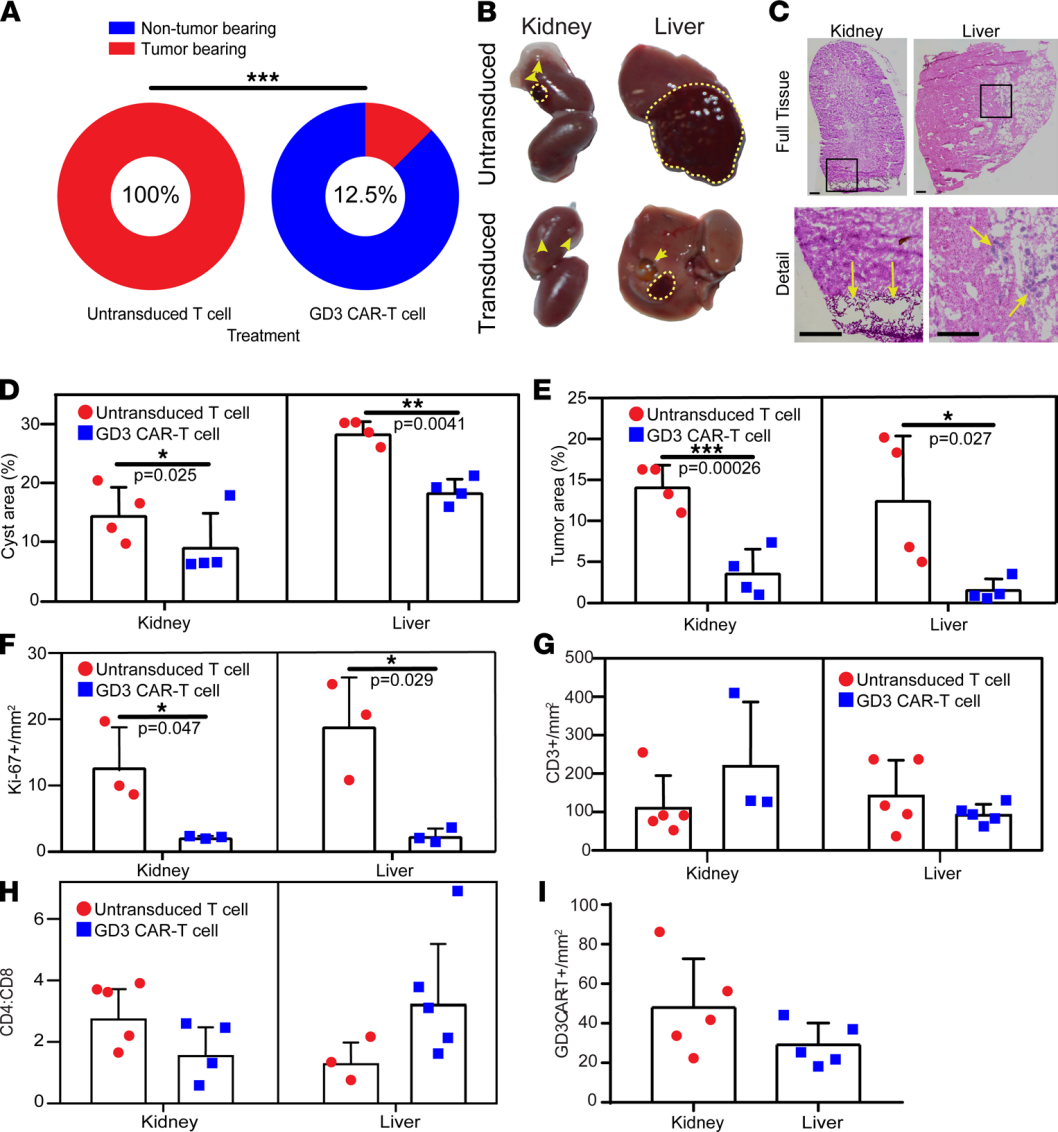
GD3 CAR T cells effectively treat spontaneous tumors arising in *Tsc2^+/–^* mice. (**A**) Tumor-bearing *Tsc2+/*^–^ mice quantified 2 weeks after initial adoptive transfer of >60-week-old mice, *n* = 10 and 8 for CAR T and untransduced T cell recipients, respectively. The presence of visible tumors was evaluated in major organs, and mice were scored as tumor positive or negative. A 2-sided Fisher’s exact test for count data was used to compare the treatment groups at *n* = 10 and *n* = 8 for the control and treatment groups, respectively. ***P = 0.0002. (**B**) Representative images of the liver and kidney tumors of the untransduced T cell recipient and GD3 CAR T cell recipient mice. Dashed lines surround tumors and arrowheads point to cysts. (**C**) Representative histology images showing cysts in the kidney and tumors in the liver of untransduced T cell recipient mice. Arrows show lesions in cross section (cysts in kidney and tumor cells in liver tissue). (**D**) Area occupied by cysts or (**E**) tumors in representative kidney and liver tissues of untransduced and GD3 CAR T cell–injected mice quantified by multiple observers (*n* = 4). One-tailed paired Student’s *t* tests were used to compare outcomes for 4 evaluators. (**F**) Ki-67–expressing cells in kidney and liver tissues quantified by immunostaining (*n* = 3). One-tailed *t* tests with unequal variance were used to compare outcomes among both treatment groups within each tissue type. (**G**) CD3^+^ T cells in available liver and kidney tissues quantified by immunostaining (*n* = 3–5). (**H**) CD4/CD8 ratios in kidney and liver tissues (*n* = 3–5) and (**I**) GD3 CAR T cells observed in representative kidney and liver tissues (*n* = 5) quantified by immunostaining. One-tailed *t* tests with unequal variance were used to compare outcomes among both treatment groups within each tissue type. *P < 0.05, **P < 0.01, ***P < 0.001. Graphs show individual values, mean values ± SD.

To evaluate the therapeutic effects of GD3 CAR T cells in vivo, we utilized aging *Tsc2+/*^–^ mice on a C57BL/6 background. These mice spontaneously develop tumors in different organs because of the biallelic mutations in the *Tsc2* gene (39). Tumors develop at an increasing frequency in liver and kidney of *Tsc2+/*^–^ mice above the age of 60 weeks. GD3 CAR T or untransduced T cells were transferred into >60-week-old *Tsc2+/*^–^ mice twice, 1 week apart, waiting another week before euthanasia. Mice were dissected and the organs were checked for remaining spontaneous tumors. Liver and kidney tissues were collected from GD3 CAR T cell– and control T cell–treated mice. We found that 8 out of 10 mice treated with GD3 CAR T cells held a significantly reduced tumor incidence compared with control mice that all exhibited tumors of the liver or kidney (Figure 6, A and B).

H&E staining and quantification of the *TSC* tumors and cysts in liver and kidney (*n* = 3–5) indicated a significant reduction in the kidney cysts and liver tumors in the GD3 CAR T cell–treated mice (Figure 6, C–E). The cell proliferation analysis based on the Ki67 staining showed a significant reduction in the number of proliferating cells in the liver and kidney of the GD3 CAR T cell–treated mice (Figure 6F and Supplemental Figure 9A). We measured T cell infiltration (CD3) to these organs (*n* = 5) (Figure 6G and Supplemental Figure 9B). The CD4/CD8 ratios in the mouse liver varied from 3.3 among CAR T cell–treated mice to 1.3 among mice treated with untransduced T cells and 1.6 to 2.8 in the mouse kidney (*n* = 4) (Figure 6H and Supplemental Figure 9, D and E). As cytotoxicity is associated with the CD8^+^ subpopulation of T cells, it appears that T cell populations in the kidney are more likely to eliminate tumor cells than T cells in the liver. The GD3 CAR T cell infiltration was detected by immunostaining and quantified in the liver and kidney tissues of the GD3 CAR T cell–injected animals (*n* = 5) (Figure 6I and Supplemental Figure 9C), with outcomes trending toward a difference in T cell subset distribution among the liver and kidney of treated animals.

To understand whether GD3 CAR T cells can mediate prolonged progression-free survival in mice, an experiment was performed using untransduced and CAR transduced T cells for adoptive transfer of immunoproficient *Tsc2+/*^–^ mice. Animals were subjected to a tumor challenge with LB1 *Tsc2*^–*/*–^ kidney tumor cells and subsequently treated with CAR transduced T cells. Here, mice initially fought off tumors, which regressed; eventually tumor growth resumed, though growth was delayed after treatment with GD3-reactive T cells (Supplemental Figure 10). These data suggest that GD3 CAR T cells can eliminate affected target cells that form *Tsc2*^–*/*–^ tumors in vivo.

To test the safety of GD3 CAR T cells, mice treated with CAR T cells were evaluated for pathologic changes on different days after adoptive transfer as shown in Supplemental Figure 11A. No pathologic changes were observed in spleen, liver, kidney, skin, lung, or brain of *Tsc2+/*^–^ mice treated by adoptive transfer. Testing was performed shortly after transfer on day 1 as well as on day 12 after transfer. Over that time frame no pathologic weight loss was observed (Supplemental Figure 11B).

Evaluating the maintenance and distribution of GD3 CAR T cells after adoptive transfer, persistence of adoptively transferred CAR T cells in several organs of recipient mice was initially predominant in the lungs, liver, spleen, and kidneys of treated mice on day 1. CAR T cells were found only in the liver on days 6 and 11 (Supplemental Figure 11C). Persistence of transferred CAR T cells improved at higher doses of 20,000–30,000 IU IL-2 (Supplemental Figure 12). Taken together the data suggest that GD3 CAR T cells can be safely applied in a preclinical model of TSC.

## Discussion

Using a combination of patient tissue analysis and functional assays performed in mouse models of the disease, our data support the therapeutic potential of GD3-reactive CAR T cells to treat benign tumors in TSC. TSC tumors overexpress GD3, similar to our prior report for LAM tissues (16). The mTOR complex controls cell growth and metabolism (40). The mTOR pathway also drives cholesterol synthesis and lipid raft formation, driving Akt signaling in a positive feedback loop supported by GD3 (41). Cholesterol itself forms a GD3 building block. Thus mTOR activation can be associated with enhanced GD3 synthesis, enhanced lipid raft formation, growth factor receptor activation, and Akt signaling (42). GD3 synthase (ST8SIA1) likely enables autophagy (43, 44) to maintain mTOR hyperactivity (45, 46) in TSC, reflecting a survival advantage of ST8SIA1^+^ cells among TSC mutant cells. This is congruent with elevated GD3 expression by *Tsc2*^–/–^ cells in response to some rapamycin concentrations (16).

Cell surface GD3 expression has likewise been considered as a target for antibody therapy of neuroendocrine tumors in small cell lung cancer (SCLC) (47). In fact, phase II clinical trials were executed, though this treatment did not become a standard of care; anti-GD3 antibodies have likewise been tested and found safe for the treatment of metastatic melanoma (48, 49).

Our current study suggests that GD3 expression in patients with TSC is not associated with high titers of anti-GD3 antibodies; lower titers were observed in patients compared with healthy controls. In aging *Tsc2* heterozygous mice, titers were not elevated either. Antibodies against GD3 can mediate cytotoxicity toward GD3-expressing cells (26). Though its significance needs to be demonstrated, having the same or lower serum antibody titers suggests limited preexisting immune surveillance at the tumor site, as we previously suggested in patients with LAM (16), hence the relevance of measuring humoral responses to the molecule.

Providing GD3-reactive antibodies by passive immunization may offer a treatment option; however, antibodies require a functional, cell-based effector mechanism or activation of complement to support antitumor activity (50). CAR T cells can overcome this limitation and, as we have shown here, can truly limit tumor growth, induce target cell death, and mediate tumor remission. Therefore, CAR T cell therapy might bring an important advance over rapamycin, which is the treatment of choice for most patients with TSC (51) or LAM (52). It will be of interest to learn how prior rapamycin treatment might affect the antitumor function of CAR T cells in patients. As rapamycin does not interfere with GD3 expression and can limit tumor growth, such treatment might be supportive (16, 53). However, rapamycin treatment can also support Treg development, which might interfere with CAR T cell function (54).

When using an amphotropic viral producer cell line, the construct can also serve to generate human GD3 CAR T cells as shown for anti-KIT designer T cells (55). Adoptive transfer of GD3 CAR T cells might fully reverse tumor development and bring long-term relief, as shown in preclinical studies of melanoma (28). This is especially true for patients with LAM, as *TSC*-knockout cells that drive tumor formation are limited, and unaffected cells are not expected to give rise to new lesions, as they might in TSC. In this respect, we previously reported consistent overexpression of GD3 in LAM lesions (16). Though we cannot rule out that cells with a single mutated *TSC1* or *TSC2* allele might also overexpress GD3, the expression patterns observed here would suggest this is not the case.

*TSC* heterozygous cells distributed over multiple organs in TSC can give rise to new lesions even after successful CAR T cell therapy. However, there are further beneficial aspects, such that patients treated by adoptive T cell therapy continue to carry a population of adoptively transferred cells even years after adoptive transfer has been performed (56). These cells are expected to become reactivated once new GD3-expressing lesions develop. Though low levels were detectable in mice followed after adoptive transfer for several days, the presence of a targetable tumor, the lymphodepletion regimen, injected CAR T cell numbers, and doses of IL-2 would influence CAR T cell clearance over time (57). In fact, it is possible that such T cells might even carry over to the next generation and be of benefit to children inheriting the same condition, as recently suggested (58). Another advantage is that patients do tolerate repeated injections of autologous T cells (59) and CAR T cells might be stored for later use once they have been generated in large numbers (60). Our data thus suggest that GD3 CAR T cell therapy might be of benefit to patients with LAM and TSC alike.

These experiments further emphasize the meaning of GD3 CAR T cell therapy for such conditions where overexpression of GD3 might be expected. Such conditions include melanoma, where benefit was demonstrated in preclinical studies (28), but also other neuroendocrine tumors, including SCLC. To this day, the latter condition has very few treatment options (61), suggesting that patients can greatly benefit from in vivo efficacy of GD3 CAR T cell treatment as supported by these data and data from other investigators.

It will be of great interest to learn whether the efficacy and longevity of GD3 CAR T cell responses will extend to newly developing lesions in different locations. The *Tsc2* heterozygous mouse model employed here displays benign tumors that develop primarily in the liver and kidneys (62). A trend toward greater infiltration by T cells and decreased CD4/CD8 in GD3 CAR T cell–treated kidneys suggests that CAR T cell efficacy is influenced by target location. Tumor-challenged models or mice with directed mutations in other organs could show whether CAR T cells will infiltrate and be effective in other body sites (63).

Polyfunctional T cells that secrete multiple cytokines sequentially or simultaneously have major implications for the efficacy of cellular therapies (64) and are used as a criterion for the assessment of treatment responses. The high polyfunctionality profile detected in the stimulated GD3 CAR T cells indicates the functional capability of these cells upon antigen encounter that will likely translate into measurable in vivo antitumor responses in patients with TSC.

Besides addressing some, but possibly not all TSC lesions that might develop in patients, the current studies may not reveal all side effects that might develop in patients. In part, this is because the actual lesions might affect different organs, but also, studies are limited by the life span of mice and by the subtle yet potentially meaningful differences in physiology (65). GD3 CAR T cells have not yet been tested in clinical settings, and the expected safety profile for GD3 CAR T cells in patients with TSC is thus based on experience with other CAR T cells from the Junghans group and others. No changes in well-being were observed in preclinical studies following adoptive transfer of GD3 CAR T cells even at the equivalent of twice the maximum dose allowable in humans (28).

Here, we identify GD3 as a TSC-associated tumor antigen, thereby overcoming a prime limitation to the design of targeted immunotherapy. *Tsc2*-deficient tumors can respond to checkpoint inhibition, suggesting that TSC is amenable to immunotherapy (66). Given the benign features of TSC, safety is an especially important aspect of treatment, and great benefit can be expected from adding an antigen-specific component to checkpoint inhibitor therapy. Spontaneous responses to growing tumors, as well as the success of targeted therapies, are clearly associated with the tumor mutation burden (67). However, TSC gives rise to monogenic tumors. We note that neoantigens are unlikely to form in tumors with mutations in only a single gene, as it takes greater than 70 de novo mutations to contribute a single neoepitope (68). Despite GD3 overexpression, tumors in TSC may thus be “cold,” providing insufficient incentive for immune infiltration and effective, spontaneous responses to mutated cells. This is congruent with the current data. At the same time, slow growth of benign tumors provides a greater window of opportunity for immunotherapy to be successful than more aggressive tumor types that are otherwise subjected to immunotherapy. Hence, our data obtained for TSC patient tissues provided the incentive to test GD3-reactive CAR T cells for the treatment of TSC in vitro and in preclinical disease models.

In all, the data presented here call for follow-up to take the application of GD3 CAR T cells to the next level and develop a clinical trial design suitable for patients with TSC. Here, we take advantage of the more predictable phenotype that results from heritable mutations giving rise to benign tumor lesions in TSC and show that GD3 expression offers a targetable antigen for CAR T cell therapy of this condition. Adoptive transfer of transgenic T cells could offer a truly new approach toward the treatment of benign tumors in TSC.

## Methods

For details, see Supplemental Methods.

### Tissue sources.

Tissues were obtained after receipt of informed consent from the National Disease Research Interchange and the National Institute of Child Health and Human Development (NICHD) Brain and Tissue Bank for Developmental Disorders at the University of Maryland (Baltimore, Maryland, USA).

### Cell culture reagents.

HEK293 (ATCC CRL-1573), HEK293-GD3 synthase (26), kidney tumor cells (LB1) from *Tsc2+/*^–^ mice (69), B16-F10 mouse melanoma cells (ATCC CRL-6475), and GD3 CAR stable viral packaging cells (28) were maintained in high-glucose DMEM supplemented with 10% FBS. Mouse T cells were cultured in complete RPMI media (Corning).

### Tissue staining.

Cryosections were stained by immunofluorescence, immunoenzymatic, or histology staining methods (70) and counterstained as relevant. Antibody information is provided in Supplemental Methods.

### Detection of humoral responses to GD3.

ELISA plates (R&D Systems) were coated with GD3 before incubation with diluted serum samples to detect anti-GD3 antibodies (16).

### Generation of GD3 CAR T cells.

T cells, purified from mouse splenocytes, were activated in culture before transduction by spinoculation using supernatant from ecotropic viral producer cells (28). Transduction was quantified by FACS.

### Fluorocytometry.

Cells were immunostained or loaded with fluorochrome as described in the Supplemental Methods and subjected to a time gate before identifying live, single cells and measuring the fluorescence among the population of interest.

### Quantification of GD3 CAR T cell–mediated cytotoxicity and cytokine production.

An IFN-γ ELISA was performed to measure cytokine secretion, and deposition of caspase-7 red dye in apoptotic cells was evaluated by IncuCyte analysis in triplicate wells to evaluate T cell function (71).

### Single-cell multiplex cytokine profiling.

To better understand individual contributions of CD4^+^ and CD8^+^ T cell subsets to CAR T cell activity and mechanism of action, CAR T cells were sorted into CD4 and CD8 subsets before coincubating each with relevant targets for 24 hours before introducing the effectors onto IsoCode chips and analyzing cytokine secretion by IsoPlexis single-cell secretome analysis (72).

### In vivo experiments with GD3 CAR T cells.

Using a tumor challenge model, LB1 *Tsc2*^–/–^ kidney tumor cells (69) were injected subcutaneously into SCID/Beige immunodeficient mice (Taconic Biosciences). Animals received 1 × 10^6^ untransduced or GD3 CAR T cells after the tumors were established. The treatment was repeated with untransduced or GD3 CAR T cells with IL-2, and tumors were measured with calipers at regular intervals. In a separate experiment, the *Tsc2+/*^–^ mice were maintained for more than 60 weeks before irradiation and adoptive transfer of untransduced or GD3 CAR T cells, supporting the T cells with intermittent IL-2 administration. Tumors were measured at endpoint. In a separate experiment, *Tsc2*^+/–^ mice (73) were challenged with tumor cells, and animals were maintained for 10 weeks after initiating adoptive T cell transfer to measure progression-free survival.

Finally, to evaluate the safety of the treatment, *Tsc2*^+/–^ mice received adoptive transfer of 5 × 10^6^ untransduced or GD3 CAR T cells. The body weight of the animals was monitored, and groups of animals were euthanized at different time points to analyze the histology of different organs.

### Statistics.

Immunostaining was quantified by ImageJ software (NIH) (74), and statistical analysis was performed using Student’s *t* test as 2-tailed tests assuming equal variance to compare TSC-affected mice or human tissues with controls. Anti-GD3 antibody titers affected and control group sera were compared by Student’s *t* test. IFN-γ cytokine release was compared at different E:T ratios and analyzed by 2-way ANOVA followed by Bonferroni’s multiple comparisons test. IncuCyte data were analyzed as apoptotic cell counts over time, and tumor growth was measured as increased tumor volumes. Both were modeled using restricted cubic splines and knots. *P* values were adjusted using a Bonferroni correction, and statistical analysis was performed using R software. Statistical analysis of the SCID/Beige tumor volume and the progression-free survival of tumor-challenged *Tsc2+/*^–^ mice was carried out using R software, splines were fit using the splines2 package, and 2-sided *P* values were calculated using a Bonferroni correction. In the spontaneous tumor model of *Tsc2+/*^–^ aged mice, a 2-sided Fisher’s exact test was applied to compare treatment groups. The *P* values are listed in the figures and figure legends. Differences were considered significant at *P* < 0.05.

### Study approval.

All animal experiments were approved by the Animal Care and Use Committee of Loyola University and Northwestern University in accordance with the current *Guide for the Care and Use of Laboratory Animals* (National Academies Press, 2011). Permission to receive human tissues from the repository was granted through Loyola University Chicago’s Institutional Review Board.

## Author contributions

ICLP developed the concept and experiments with consultation from DFD, and AT, RPJ, SS, ERD, and ICLP contributed to the interpretation of the results and writing of the manuscript with input from all coauthors. AT, SA, and JZP performed in vivo experiments. ERD, SS, ZM, NG, SRZ, SWH, PR, RSS, DJ, JOZ, and AT performed and evaluated staining. RSS measured anti-GD3 titers and antibody-mediated cytotoxicity, and LB and FH generated the LB1 *Tsc2^–/–^* tumor cell line. CCV, NL, and DMS analyzed the tumor challenge and cytotoxicity data. ASYL supplied rabbit anti-GD3 antibody and GD3 CAR construct with RPJ. MMP, TND, JM, DFD, and RPJ provided and evaluated critical tissues and other materials.

## Supplementary Material

Supplemental data

## Version 1 11/22/2021

Electronic publication
