# Supplementary material for: Benign tumors in TSC are amenable to treatment by GD3 CAR T cells in mice
Source: JCI Insight. 2021 Nov 22;6(22):e152014. doi: 10.1172/jci.insight.152014 (PMC8663788; doi:10.1172/jci.insight.152014)
Supplement: Supplemental data [file jciinsight-6-152014-s084.pdf]

Supplement:

Materials and Methods

Tissue Sources

Adult human brain, kidney, skin, and lung tissues from consented TSC patients were obtained from the National Disease Research Interchange (NDRI) and the National Institute of Child Health and Human Development (NICHD) Brain and Tissue Bank for Developmental Disorders at the University of Maryland. Additional TSC lesional and nonlesional skin sections were acquired from the Pulmonary Branch, National Heart, Lung, and Blood Institute, National Institutes of Health (Bethesda, MD). Normal human brain, kidney, and lung tissues were also obtained from the National Disease Research Interchange (NDRI) and as otherwise discarded, operative material from Loyola University's Department of Pathology (LUMC).

All tissue procurement was approved by Loyola University's Institutional Review Board and followed the Declaration of Helsinki adopted by the World Health Organization. Samples were snap frozen in optimal cutting temperature (OCT) compound (Leica-CM3050S) on site or shipped from NDRI and stored at -80ºC.

TSC patient sera were obtained from NDRI, and normal sera were donated by healthy volunteers.

Cell Culture Reagents

HEK293 (ATCC CRL-1573), HEK293-GD3synthase (1), kidney tumor cells (LB1) from *Tsc2*$^{+/-}$ mice (2), B16-F10 mouse melanoma cells (ATCC CRL-6475), and GD3 CAR stable viral packaging cells (3) were maintained in high-glucose DMEM media supplemented with 10% FBS and antibiotic/antimycotic (all from Gibco-Invitrogen). Mouse T cells were cultured in RPMI media supplied with 10% FBS, 1X non-essential amino acids (Corning), 50 U/ml penicillin-streptomycin (Thermo Fisher Scientific), 1 mM sodium pyruvate (Gibco), 10 mM HEPES (Gibco), 50 μM β-mercaptoethanol (Sigma-Aldrich).

Tissue staining

Frozen tissues sections (8 µm) were cut using a cryostat (Leica CM 3050S). Cryosections were fixed in cold acetone for 10 minutes and stored at -20°C as needed, followed by immunostaining and enzymatic development. Sections were blocked using Super Block (SkyTek Labs) and where needed, 5% rat serum was used as well. Some tissues were formalin fixed, paraffin embedded and sectioned (6 µm).

For human tissues, primary antibodies were used at empirically determined concentrations in 10% normal human serum (Gemini Bioproducts) in buffer for both IF and IHC, including antibody to GD3 (R24) (Absolute Antibody) and anti-GD3 synthase (K-18) (Santa Cruz Biotech) to detect cells expressing the antigen of interest. Anti-phospho-S6 S235/236 antibody (clone) (Cell Signaling Technology) was used to identify cells with hyperactive mTOR (4) and anti-CD3 (UCHT1) (BD Biosciences) to locate T cells. PE-labeled MEM-188 to CD56 (Biolegend) was used alone or combined with anti-CD3 to identify NK and NKT cells, respectively. Antibody 6B11 to the invariant vα24-JαQ TCR chain (Biolegend) was used to identify iNKT among NKT cells. Antibody 51.1 to CD1d (Biolegend) was used in combination with antibody 3.9 to CD11c (Biolegend) to locate cells capable of presenting the GD3 antigen to NKT cells (1). For enzymatic staining, peroxidase labeled goat anti-mouse secondary antibody (#1100-05 Southern Biotech) was used. Texas Red labeled goat anti-mouse antibodies (#T6930 Invitrogen), and AF488 labeled goat anti-rabbit (#A-21070 Thermo Scientific) were used for fluorescent detection. Biotinylated goat anti-mouse antibody (#1100-08 Southern Biotech) was detected using FITC-labeled streptavidin (#7100-02 Southern Biotech).

Mouse tissues were harvested after euthanasia and stored as frozen or paraffin embedded tissues. For the *TSC* cyst and tumor quantifications, standard hematoxylin and eosin (H&E) sections were prepared from mouse kidneys and liver tissues after acetone fixation. Quantification of kidney cystadenomas and liver hemangioma were done by four independent observers, covering a full tissue section. Cyst areas enclosing greater than 10,000 μm^2 were quantified as a percentage of total area, and tumor burden was quantified as a percentage of total area as described (5, 6) with slight modifications. Immunofluorescence staining was performed using PE-labeled anti-CD3 antibody (145-2C11), AF647-labeled anti-CD4 (L3T4), anti-CD8a (53-6.7), and anti-Ki67 (16A8) purchased from Biolegend. AF647-labeled donkey anti-rabbit (#406414 Biolegend) was used as a secondary antibody to detect CAR

expression or rabbit primary antibody to GD3 in tissue sections. Sections were 4′,6-diamidino-2-phenylindole (DAPI) counterstained (BD Biosciences) to identify and count nucleated cells. Stained sections were imaged using a Revolve microscope (Discover Echo Inc.) for enzymatic and H&E staining and, Revolve or Ti2 Widefield (Nikon) microscopes for fluorescence-based whole slide evaluation.

Detection of humoral responses to GD3

GD3 ELISAs were performed as reported (7) by coating the ELISA plate (R&D Systems) with GD3 antigen (Enzo Lifesciences) at 5ng/ml in methanol and allowing the solvent to evaporate overnight. The plate was blocked using PBS with 0.05% Tween and 0.1% bovine serum albumin (BSA) for 1 hour at room temperature. Sera was pooled either from six healthy subjects or from fourteen TSC patients and then diluted in PBS. Bound anti-GD3 antibodies were detected using horseradish peroxidase-labeled goat anti-mouse or anti-human IgG3 (Southern Biotech). Wells were subsequently incubated with equal concentrations of stabilized hydrogen peroxide and tetramethylbenzidine substrate (R&D Systems) and the reaction was stopped using 0.67N sulfuric acid. Reaction product was measured in a Polar Star Omega ELISA plate reader (BMG Labtech) at 450 nm. Data were analyzed using MARS data analysis software (BMG Labtech).

Generation of GD3 CAR T-cells

The second generation GD3 CAR construct was generated and validated earlier (3). Total T cells were isolated from spleens of 12-20 weeks old *Tsc2*$^{+/+}$ mice using EasySep mouse T cell isolation kits (Stemcell Technologies) following the manufacturer's instructions. T cells were activated using anti-CD3/CD28 Dynabeads (Thermo Fisher Scientific) at a 1:1 bead to cell ratio and 30 IU/ml rhIL-2 for 24 hours before transduction. Twenty-four well non-tissue culture plates were coated with 10 µg/ml retronectin (Takara) and supernatant of near-confluent GD3 CAR ecotropic vector producer cells was spun onto the retronectin-coated plate. Activated T cells (10^6/well) were transferred to the plate with additional viral supernatant plus 5 mg/ml protamine sulfate (Sigma Aldrich) and 30 IU/mL rhIL-2 (Hoffmann La Roche and NIH/NCI biological resources branch, Bethesda, MD). Cells were spinoculated at 1,000 x g for 2 hours, followed by overnight incubation at 37°C in complete medium with 30 IU/ml rhIL-

2. The transduction was repeated once, the next day. Transduced T cells were reactivated with anti-CD3/CD28 beads at 1:1 bead to cell ratio and expanded in complete RPMI with 10% fetal bovine serum (FBS) containing 30 IU/mL rhIL-2 for 2 days.

Fluorocytometry

For flow cytometry using the BD Symphony (Becton Dickenson) cells were gated on single and live CD3 positive cells. GD3 CAR expression was quantified on CD4 and CD8 T cells. The rabbit polyclonal antibody to GD3 CAR construct was made and validated previously. This construct encodes a second generation GD3 CAR construct fused with a short fragment of extracellular, transmembrane, and intracellular sequences of CD28 plus the intracellular functional domain of the CD3 zeta chain (3). FITC-labeled anti-CD3 (BD Biosciences), BV421-labeled anti-CD4 (BioLegend), AF594-labeled anti-CD8 (BioLegend), and APC-labeled goat anti-rabbit antibodies (#A10931 Invitrogen) and near-IR fluorescent reactive dye (Thermo Fisher Scientific) were used for flow analysis.

The in vitro expansion of transduced and untransduced T cells was compared after T transduction. T cells were left to recover overnight with CD3/CD28 coated beads and IL-2 in complete T cell medium. One μM carboxyfluorescein succimidyl ester (CFSE) was used to label the T cells before returned the cells to media with activation beads and IL-2 for 6 days, taking samples of the population for FACS analysis beforehand and on days 2, 4 and 6. Also, the total T cell count was monitored over 10 days.

Quantification of GD3 CAR T-cell mediated cytotoxicity and cytokine production

To measure IFNγ secretion as a functional readout of T cell activity, the cytokine was quantified by ELISA. Fifty thousand target cells (LB1) *Tsc2*$^{-/-}$ tumor cells or HEK293-GD3synthase^{+} cells) were plated with untransduced or GD3 CAR T-cells at 3 different effector: target (E:T) ratios (100 - 1:1). Co-cultures were seeded in triplicate and incubated for 48 hours. Supernatant were stored at -20°C to be used in a mouse IFNγ ELISA (R&D Systems) performed according to the manufacturer’s protocol.

Cytotoxicity mediated apoptosis by GD3 CAR T- cells was measured by adding 1 μM caspase-7 red dye (Essen Bioscience) and acquired live images of red cells that have undergone caspase-3/7

mediated apoptosis. to 96-well coated plates and imaging red fluorescence over time using the Incucyte S3 system (Essen Bio Science) (8). Briefly, 3,000 target cells were seeded in a flat bottom 96-well plate overnight. Untransduced or GD3 CAR transduced mouse T cells were added to LB1 *Tsc2*$^{-/-}$ tumor cells (E:T-10:1) or HEK293-GD3 synthase^{+} target cells in different E:T ratios (10 - 0.1:1) in triplicate and incubated for 48 hrs. Three images/well were acquired every 3 hours for 48 hours and apoptotic cells stained in red were quantified using Incucyte S3 software (Essen Bioscience).

Single-cell multiplex cytokine profiling

T cells isolated from *Tsc2*$^{+/+}$ mouse spleen, untransduced or transduced with the GD3 CAR construct were recovered from liquid nitrogen in complete RPMI with IL-2 (100 IU/ml) overnight. Dead cells were removed using mouse lymphocyte cell separation media (Cedarlane) and the CD4^{+} CAR or untransduced T cells were enriched using anti-CD4 (L3T4) microbeads (Miltenyi Biotec) using a LS column in a MACS separator (Miltenyi). The positively selected CD4 fraction was eluted, and unbound cells represented the CD8^{+} T cells. Both subsets were individually co- cultured with GD3 expressing HEK293 cells (1) for 20 hrs in a 6-well plate at an E:T ratio of 1:2.

After stimulation, the T cells were collected and stained with membrane stain A before loading T cells onto a single cell polyfunctional strength mouse Isocode Chip (IsoPlexis) pre-patterned with a 28-plex antibody array (9). Cells were imaged to identify wells with single cells and the assay was run at 37 °C, 5% CO2 for 24 h in an IsoPlexis cytokine profiler. The secretory profiles of single cells were analyzed by IsoSpeak Software (IsoPlexis) independently detecting 28 different analytes secreted by live mouse T cells (10-12).

In vivo experiments with GD3 CAR T-cells

The tumor challenge model employed here engaged *Tsc2*$^{-/-}$ cells derived from the *Tsc2*$^{+/-}$ mice that developed spontaneous tumors in multiple sites (13). Kidney tumor tissue was surgically isolated from a 21-month-old male *Tsc2*$^{+/-}$ mouse (The Jackson Laboratory) and mechanically digested, washed in Hanks Balanced Salt Solution (Invitrogen) and plated in serum-supplemented Dulbecco's

Modified Eagle's Medium (Invitrogen). After several passages, loss of *TSC2* expression was confirmed by western blot as described (14).

The *Tsc2*$^{-/-}$ (LB1) tumor cells were confirmed to be pathogen free (Charles River Laboratories) before subcutaneous injection of 10^6 cells into 14-18 week-old male and female C.B-*Igh*-1b/GbmsTac-*Prkdc*^{scid}-*Lyst*^{bg} N7 SCID/bBeige mice (Taconic Biosciences). Three weeks after tumor inoculation, animals received 10^6 live untransduced or >70% GD3 CAR T-cells (15). T cells were supported by 5,000 IU/mouse rhIL-2 (Hoffmann La Roche and NIH/NCI biological resources branch, Bethesda, MD) intraperitoneally injected every other day. Two additional T cell adoptive transfers were performed at 1-week intervals. Tumor growth was followed throughout the experiment. Mice were euthanized and tissues were collected for analysis a week after the last adoptive transfer procedure.

For in vivo experiments with the *Tsc2*$^{+/-}$ spontaneous tumor model, we employed male and female B6.129S4-*Tsc2*^{tm1Djk}/J mice (Jackson Laboratory) that were intercrossed for maintenance. These experiments complement the adoptive transfer data from immunodeficient mice to reveal how CAR T cells function in an immunoproficient environment. Offspring included in experiments were genotyped to verify heterozygosity for *Tsc2.* To await consistent spontaneous tumor development, these mice were included in experiments beyond 60 weeks of age. Mice were administered with 10^6 live >70% GD3 CAR transduced or untransduced T-cells by retro-orbital injection. Mice in both groups received an additional adoptive T- cell transfer after 1 week. On the day of transfer and every other day until termination all mice received 50,000 IU rIL-2 by IP injection as above to support T cell function in these mice. Mice were euthanized and tissues were collected for analysis 1 week after the second adoptive transfer of T cells. Tumor incidence was evaluated at euthanasia. At that time, blood was drawn for serum and tumor-bearing tissues were collected and photographed.

In order to test the progression free survival in tumor-challenged Tsc2 $^{+/-}$ mice, animals were subcutaneously challenged with 2.5x 10^6 *Tsc2*$^{-/-}$ (LB1) tumor cells. After 3 weeks, mice were lymphodepleted by 5 Gy irradiation and treated with 3 weekly injections of 10^6 untransduced or GD3 CAR-T cells or PBS. All mice received 30,000 IU of IL-2 IP 3 times per week. Tumor growth was followed for 10 weeks after treatment initiation. To evaluate the safety of the treatment, mice received adoptive transfer of 5X10^6 untransduced or GD3 CAR T-cells. Mice were weighed and euthanized at regular

intervals until 11 days after T cell transfer. Tissues were harvested in part for paraffin embedding and H&E staining, and in part to analysis of GD3 CAR T-cell infiltration and persistence through flow cytometry.

Data analysis and statistics

Staining was quantified per number of cells or per area using Image J software (16). Student's t-test was used where TSC-affected tissues, serum titers and CAR-T cell treatments compared to controls, and bars show mean values +/- SD. Unless otherwise specified, throughout the manuscript t-tests were performed as two-tailed tests assuming equal variance.

To examine the treatment effect on counts of apoptotic cells over time through IncuCyte live-cell imaging, a generalized linear mixed model (GLMM) with log link and zero-inflated quasi-Poisson distribution assumption was used and included fixed effects for treatment group and a random effect for well. An unstructured covariance structure for repeated measures was used and we examined several distributions including Poisson, Zero-Inflated Poisson, Zero-Inflated Negative Binomial, and Zero-Inflated quasi-Poisson models. Apoptotic cell count by time was modelled using restricted cubic splines with three knots set at equally spaced quantiles (12, 24, and 36 hours) to flexibly model the association. Pairwise comparisons of treatment on log of expected count of apoptotic cells were conducted and p-values were adjusted using a Bonferroni correction for multiple testing. Statistical analyses were carried out using R version 3.6.2 software (17). Restricted cubic splines were estimated with the rms package (18) and the GLMM model was fit using the glmmTMB package (19). Differential IFNγ release was compared in a two-way analysis of variance (ANOVA).

To examine treatment effect and group differences in tumor challenge experiments, tumor volume over time was modeled using cubic splines with knots set at day of treatment injection (day 8, day 15). Polynomials were fitted within intervals set by knots and restrictions were placed on the resulting curve to ensure a smooth appearance at the knot points, to provide a flexible way of estimating the underlying regression function without assuming a linear trend. Models included treatment effects with a reference spline term and interaction terms between treatment and spline terms. Likelihood ratio tests were used to evaluate statistical significance of the treatment by spline interaction terms. Statistical analysis was again carried out using R software and splines were fit using the splines2 package. All reported *p*-values were

two sided. A Bonferroni correction was used such that individual comparison critical values should have a *p*-value < 0.0167 to be considered statistically significant.

In the *Tsc2*$^{+/-}$ spontaneous tumor model, two-sided Fisher's Exact Test for count data was used compare the treatment effects in between the two experimental groups. In all the analyses, p-values are shown as * p<0.05, ** p<0.005, *** p<0.0005.

References

1. Eby JM, Barse L, Henning SW, Rabelink MJ, Klarquist J, Gilbert ER, et al. Alpha-N-acetyl-neuraminide alpha-2,8-sialyltransferase 1 can support immune responses toward tumors overexpressing ganglioside D3 in mice. *Cancer Immunol Immunother.* 2017;66(1):63-75.
2. Han F, Dellacecca ER, Barse LW, Cosgrove C, Henning SW, Ankney CM, et al. Adoptive T-Cell Transfer to Treat Lymphangioleiomyomatosis. *Am J Respir Cell Mol Biol.* 2020;62(6):793-804.
3. Lo AS, Ma Q, Liu DL, and Junghans RP. Anti-GD3 chimeric sFv-CD28/T-cell receptor zeta designer T cells for treatment of metastatic melanoma and other neuroectodermal tumors. *Clin Cancer Res.* 2010;16(10):2769-80.
4. Dufner A, and Thomas G. Ribosomal S6 kinase signaling and the control of translation. *Exp Cell Res.* 1999;253(1):100-9.
5. Pollizzi K, Malinowska-Kolodziej I, Stumm M, Lane H, and Kwiatkowski D. Equivalent benefit of mTORC1 blockade and combined PI3K-mTOR blockade in a mouse model of tuberous sclerosis. *Mol Cancer.* 2009;8:38.
6. El-Hashemite N, Walker V, and Kwiatkowski DJ. Estrogen enhances whereas tamoxifen retards development of Tsc mouse liver hemangioma: a tumor related to renal angiomyolipoma and pulmonary lymphangioleiomyomatosis. *Cancer Res.* 2005;65(6):2474-81.
7. Gilbert ER, Eby JM, Hammer AM, Klarquist J, Christensen DG, Barfuss AJ, et al. Positioning ganglioside D3 as an immunotherapeutic target in lymphangioleiomyomatosis. *Am J Pathol.* 2013;183(1):226-34.

8. Granger JE, and Appledorn DM. Kinetic Measurement of Apoptosis and Immune Cell Killing Using Live-Cell Imaging and Analysis. *Methods Mol Biol.* 2021;2255:197-212.

9. Liu D, Paczkowski P, Mackay S, Ng C, and Zhou J. Single-Cell Multiplexed Proteomics on the IsoLight Resolves Cellular Functional Heterogeneity to Reveal Clinical Responses of Cancer Patients to Immunotherapies. *Methods Mol Biol.* 2020;2055:413-31.

10. Xue Q, Bettini E, Paczkowski P, Ng C, Kaiser A, McConnell T, et al. Single-cell multiplexed cytokine profiling of CD19 CAR-T cells reveals a diverse landscape of polyfunctional antigen-specific response. *J Immunother Cancer.* 2017;5(1):85.

11. Parisi G, Saco JD, Salazar FB, Tsoi J, Krystofinski P, Puig-Saus C, et al. Persistence of adoptively transferred T cells with a kinetically engineered IL-2 receptor agonist. *Nat Commun.* 2020;11(1):660.

12. Anagnostou V, Bruhm DC, Niknafs N, White JR, Shao XM, Sidhom JW, et al. Integrative Tumor and Immune Cell Multi-omic Analyses Predict Response to Immune Checkpoint Blockade in Melanoma. *Cell Rep Med.* 2020;1(8):100139.

13. Goncharova EA, Goncharov DA, Fehrenbach M, Khavin I, Ducka B, Hino O, et al. Prevention of alveolar destruction and airspace enlargement in a mouse model of pulmonary lymphangioleiomyomatosis (LAM). *Science translational medicine.* 2012;4(154):154ra34.

14. Han F, Dellacecca ER, Barse LW, Cosgrove C, Henning SW, Ankney CM, et al. Adoptive T Cell Transfer to Treat Lymphangioleiomyomatosis. *Am J Respir Cell Mol Biol.* 2020.

15. Eby JM, Smith AR, Riley TP, Cosgrove C, Ankney CM, Henning SW, et al. Molecular properties of gp100-reactive T-cell receptors drive the cytokine profile and antitumor efficacy of transgenic host T cells. *Pigment cell & melanoma research.* 2019;32(1):68-78.

16. Klein C, Devi-Marulkar P, Dieu-Nosjean MC, and Germain C. Development of Tools for the Selective Visualization and Quantification of TLS-Immune Cells on Tissue Sections. *Methods Mol Biol.* 2018;1845:47-69.

17. R Core Team , R: A language and environment for statistical computing. *R Foundation for Statistical Computing.* 2019.

18. Harrell Jr FE. rms: Regression Modeling Strategies. *R package version 51-4.* 2019.

19. Brooks ME, Kristensen K, Benthem KJv, Magnusson A, Berg CW, Nielsen A, et al. glmmTMB Balances Speed and Flexibility Among Packages for Zero-inflated Generalized Linear Mixed Modeling. *The R Journal.* 2017;9(2):378-400.

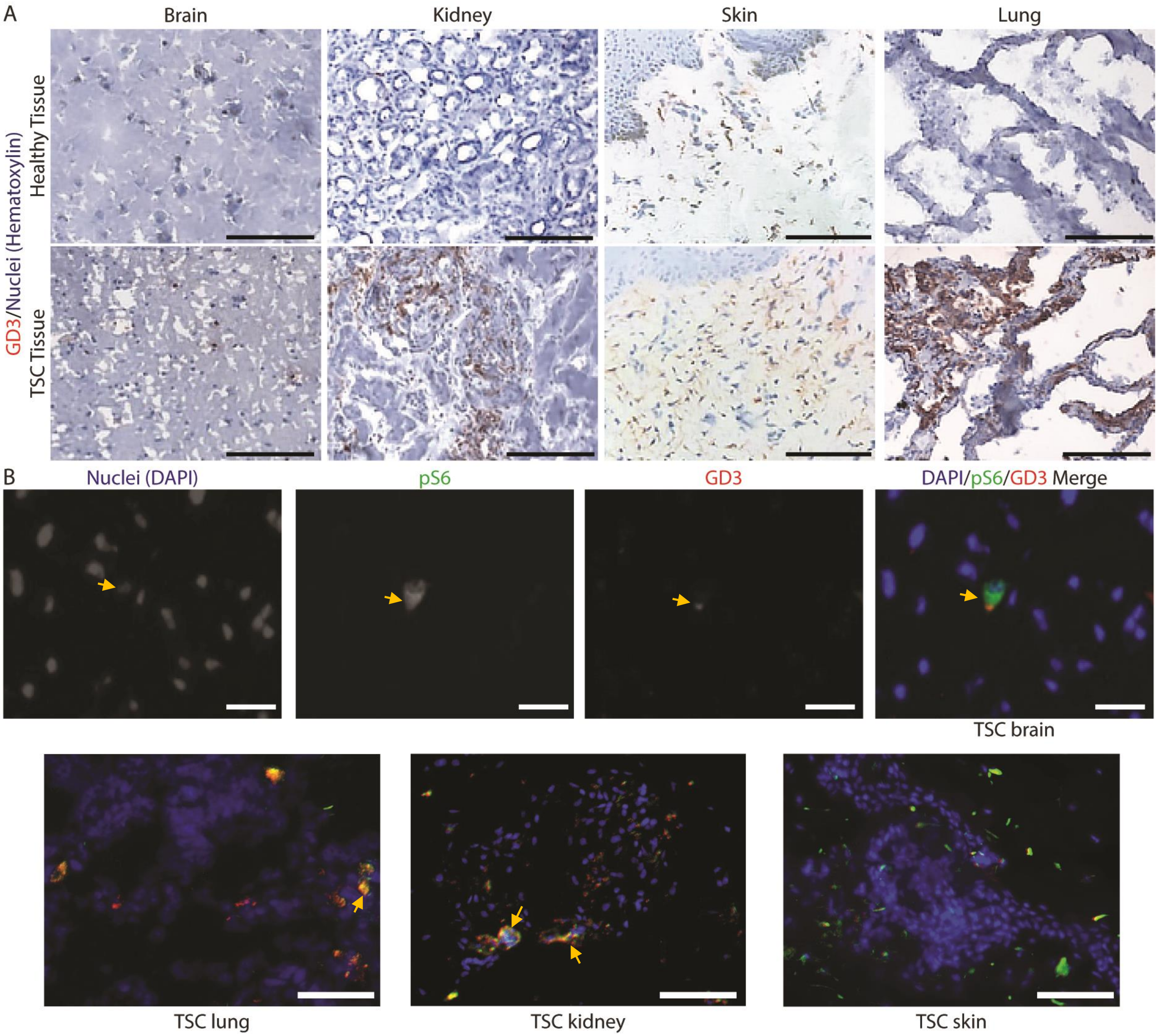

Supplementary FigureS1: **Tissue immunohistology in TSC.** A) Representative images of GD3 expression in TSC and healthy tissues; scale bar= 50 µm. (B) Co-localization of GD3 (Texas red) and phospho-S6 (Alexa Fluor 488) staining in a TSC brain tissue sample and example double staining of TSC lung, kidney and skin. Scale bar= 50µm, yellow arrows indicate double-stained cells.

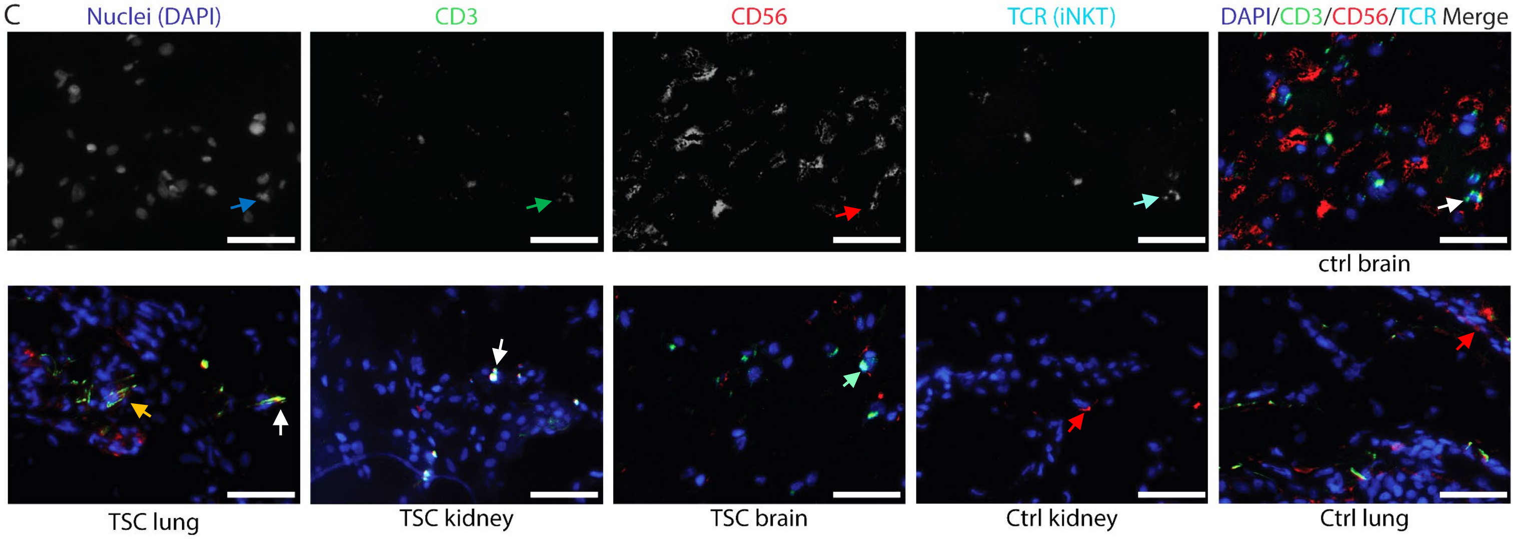

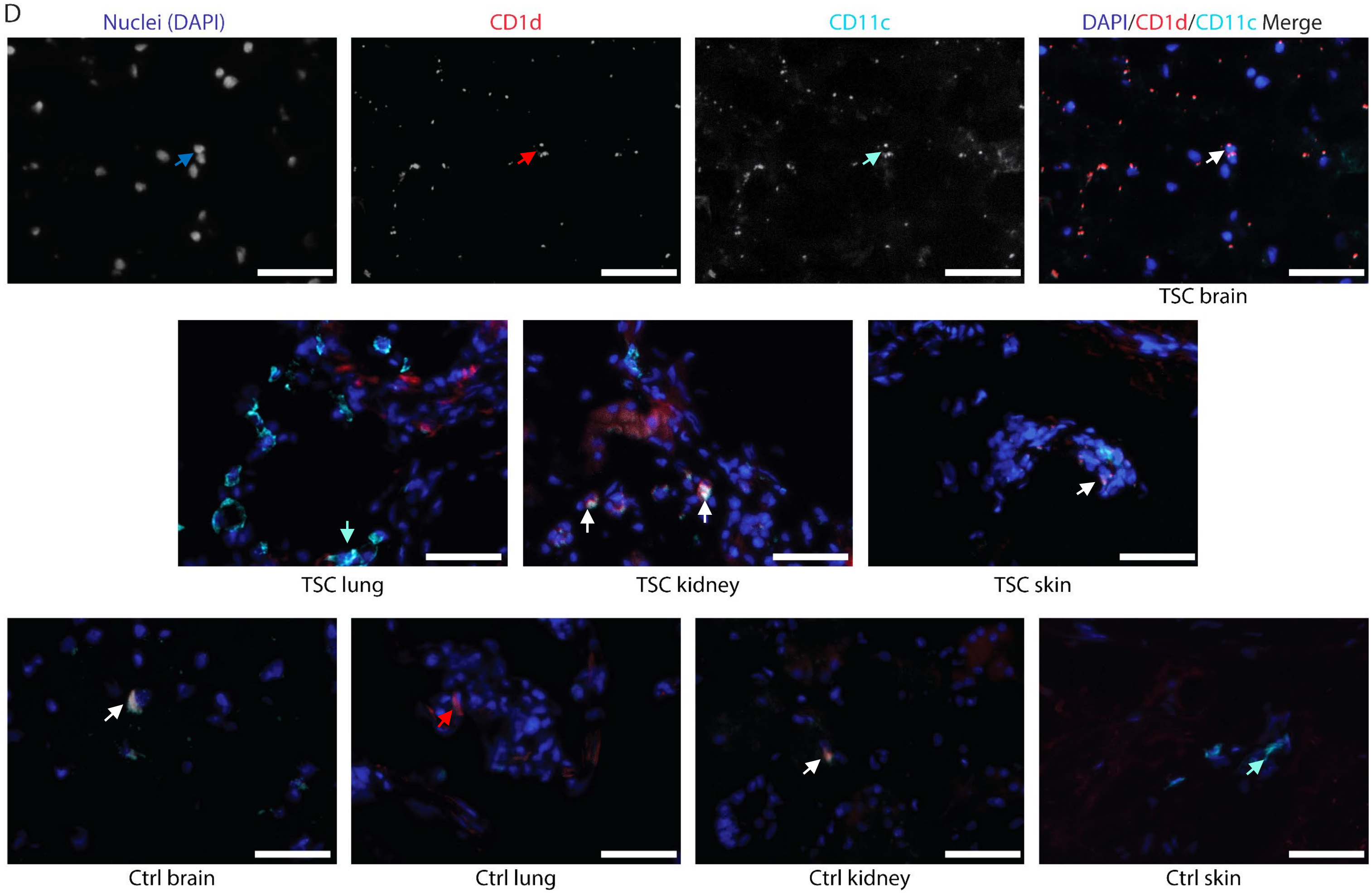

Supplementary FigureS1, continued: Tissue immunohistology in TSC. (C) Locating iNKT cells though invariant TCR expression, CD3 and CD56 co-staining in TSC tissues. (D) CD1d-expressing DCs (CD1d-CD11c) in TSC and control tissues. scale bar =50μm, arrows indicate examples of stained cells in corresponding colors.

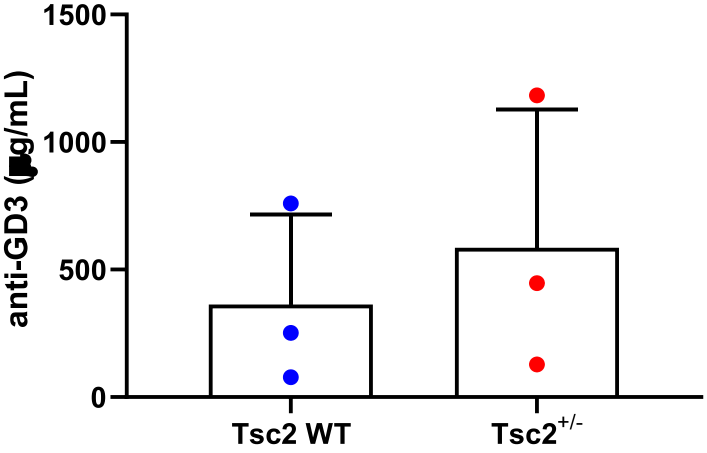

Supplementary Figure S2: Anti-GD3 serum titers are not elevated in a mouse model of TSC. Sera were was harvested from aged (10+ months) *Tsc2*$^{+/-}$ mice (n=3) and from C57BL/6 (n=3) mice, and anti-GD3 titers were measured by ELISA. Briefly, plates were coated with GD3 before adding sera and detecting anti-GD3 antibodies using peroxidase-labeled horse anti-mouse IgG antibodies. ELISA plates were developed with TMB substrate. The two groups were compared by a two-tailed student's t-test assuming equal variance and no significant difference was detected.

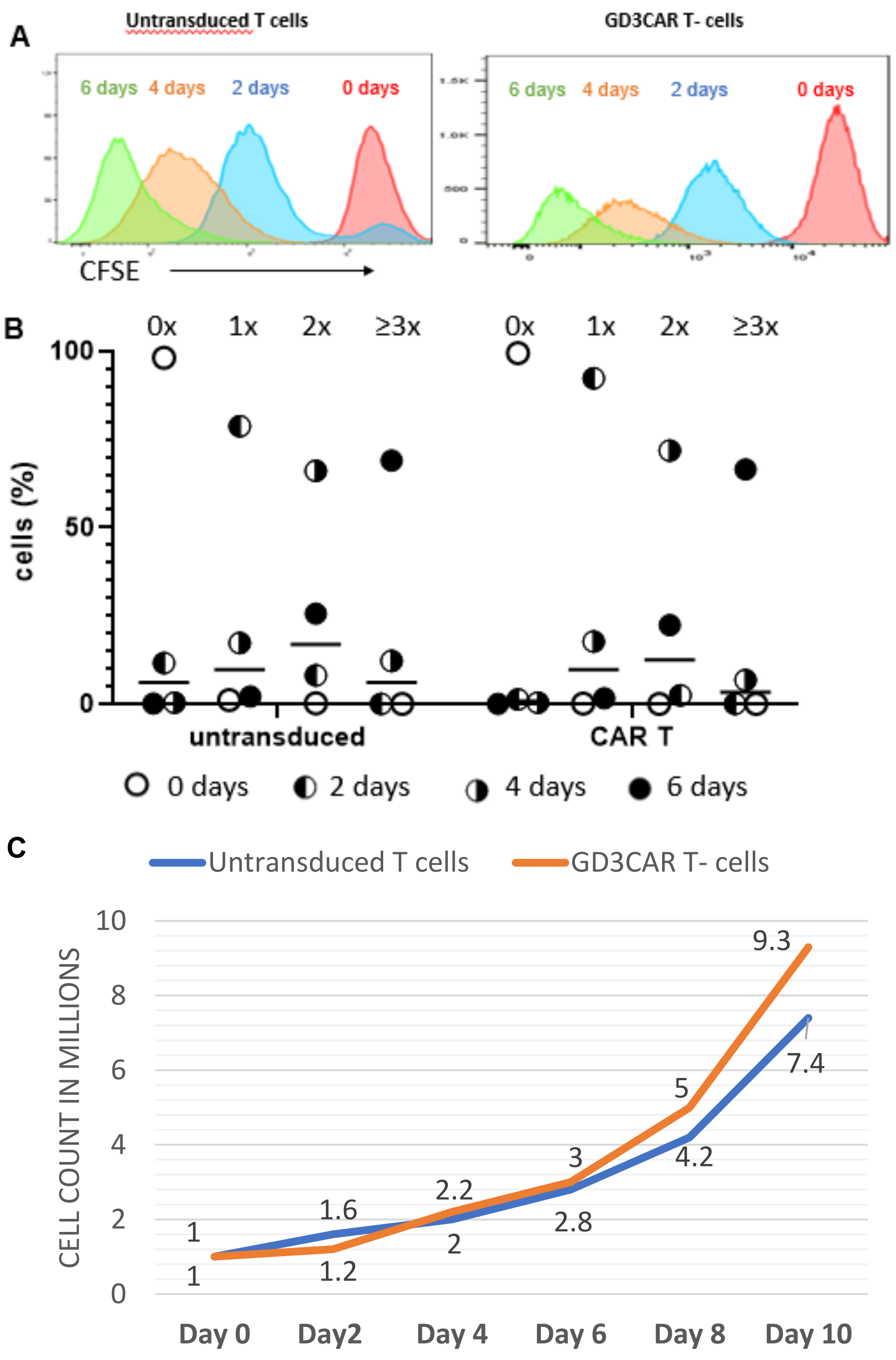

Supplementary Figure S3: In vitro expansion of GD3 CAR T-cells. T cell expansion is not impaired by GD3CAR transduction. Untransduced and CAR transduced mouse T cells were recovered overnight with CD3/CD28 coated beads and IL-2 in complete T cell medium before adding 1uM CFSE for 5 minutes. Cells were washed and returned to media with activation beads and IL-2 for 6 days. (A) Measurement of fluorescence from the samples of the populations after staining and on days 2, 4 and 6 to by FACS. (B) CFSE dilution was evaluated as a measure of proliferation and quantified as 0x, 1x, 2x and 3x or more cell divisions. Proliferation among CAR T cells was not reduced compared to untransduced T cells. (C) The total T cell count over 10 days for untransduced and GD3 CAR T-cells. In figures B and C, data were analyzed by two-way ANOVA and no differences were found among T- cell types or days.

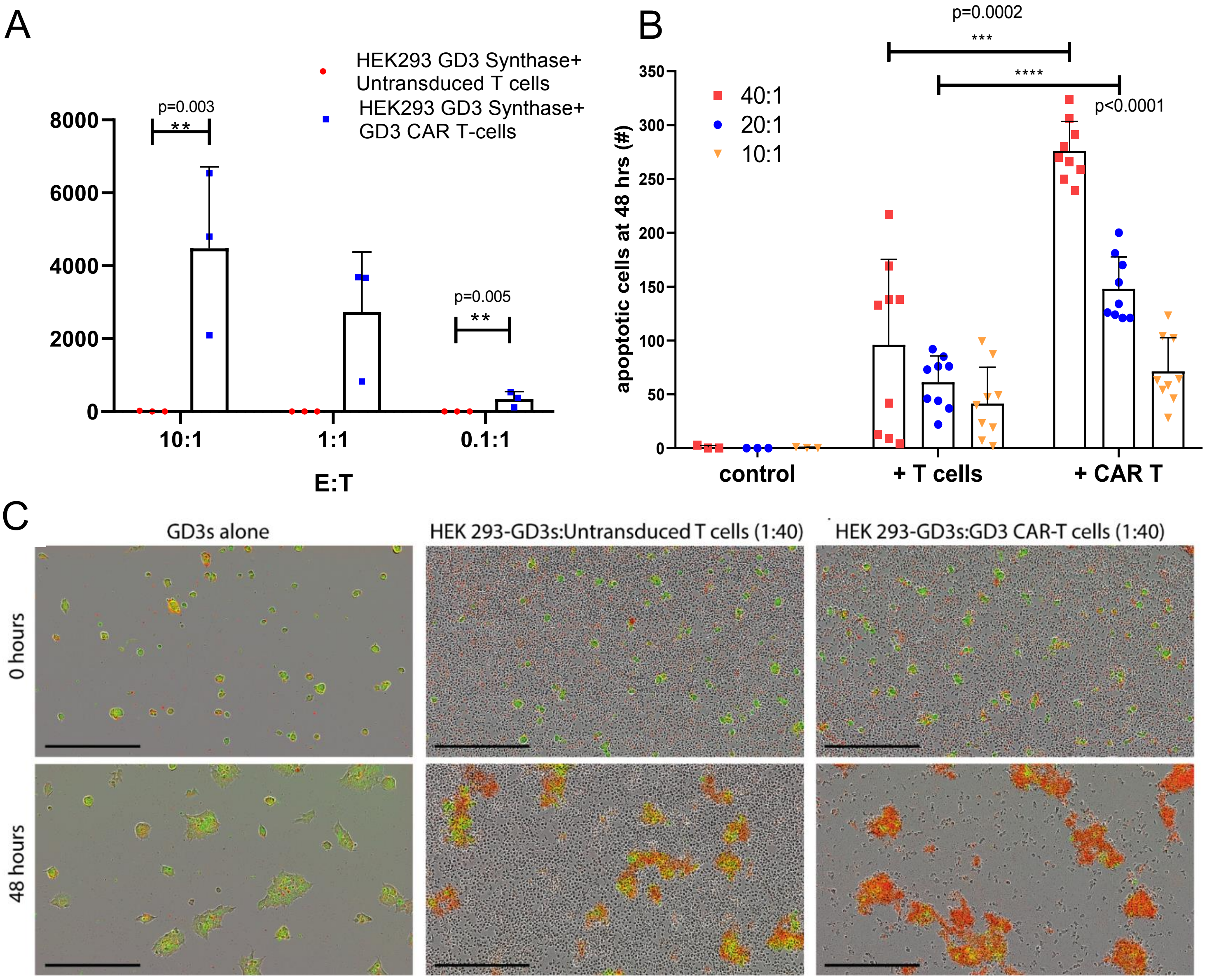

Supplementary Figure S4: Mouse CAR T-cells react with GD3 synthase transduced HEK293 target cells. (A) IFNγ secretion by GD3 CAR T-cells in response to GD3-overexpressing HEK293 cells. Results combined from 3 individually performed experiments, analyzed by two-way ANOVA followed by Tukey's multiple comparisons test. Differences at 10:1 and 0.1:1 E:T ratios were significant at **p=0.003 and 0.005, respectively. (B) The number of apoptotic cells was quantified in wells plated with 3000 target cells/well, adding untransduced T cells or CAR T cells at E:T ratios of 10, 20 and 40:1 for 48 hrs., comparing outcomes by two-way ANOVA followed by Tukey's multiple comparisons test. CAR T cells displayed significant cytotoxicity at every E:T ratio and cytotoxicity was also increased when comparing CAR T to untransduced T cells at 40:1 (2.9-fold, p=0.0002) or 20:1 (2.4-fold, p<0.0001). (C) Representative images of target cell death (red) induced by GD3 CAR T-cells and untransduced T cells, scale bar = 400 μm.

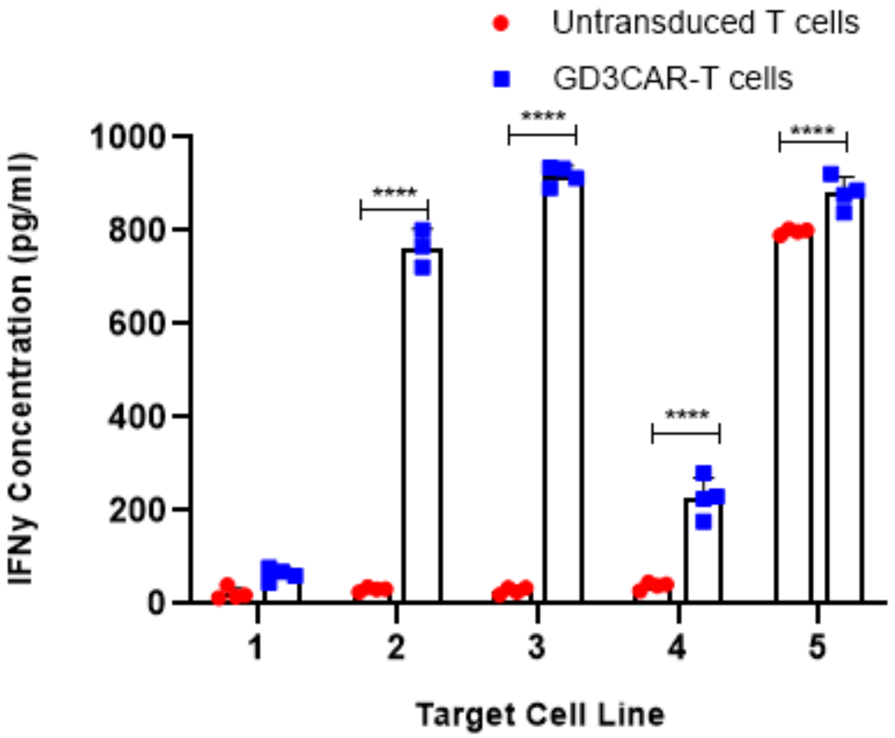

Supplementary Figure S5: IFNγ secretion by GD3 CAR-T cells in response to GD3-overexpressing HEK293 and human melanoma cells. GD3 CAR transduced mouse T cells were co-cultured 1:1 with various target cells, 1. HEK293, 2. HEK293 + human GD3 synthase, 3. human melanoma cells-624.38, 4. Unstimulated T cells alone, and 5. PMA/ionomycin stimulated T cells. T cell activation was measured by ELISA as IFNγ released into the supernatant in triplicates and analyzed by performing two-way ANOVA (****$p<0.0001$ for target cells and T cell types) followed by Sidak's multiple comparison test. Bar graphs show mean values +/- SD. For all p values shown, ****$p<0.0001$. The figure shows representative data from one experiment of the three performed.

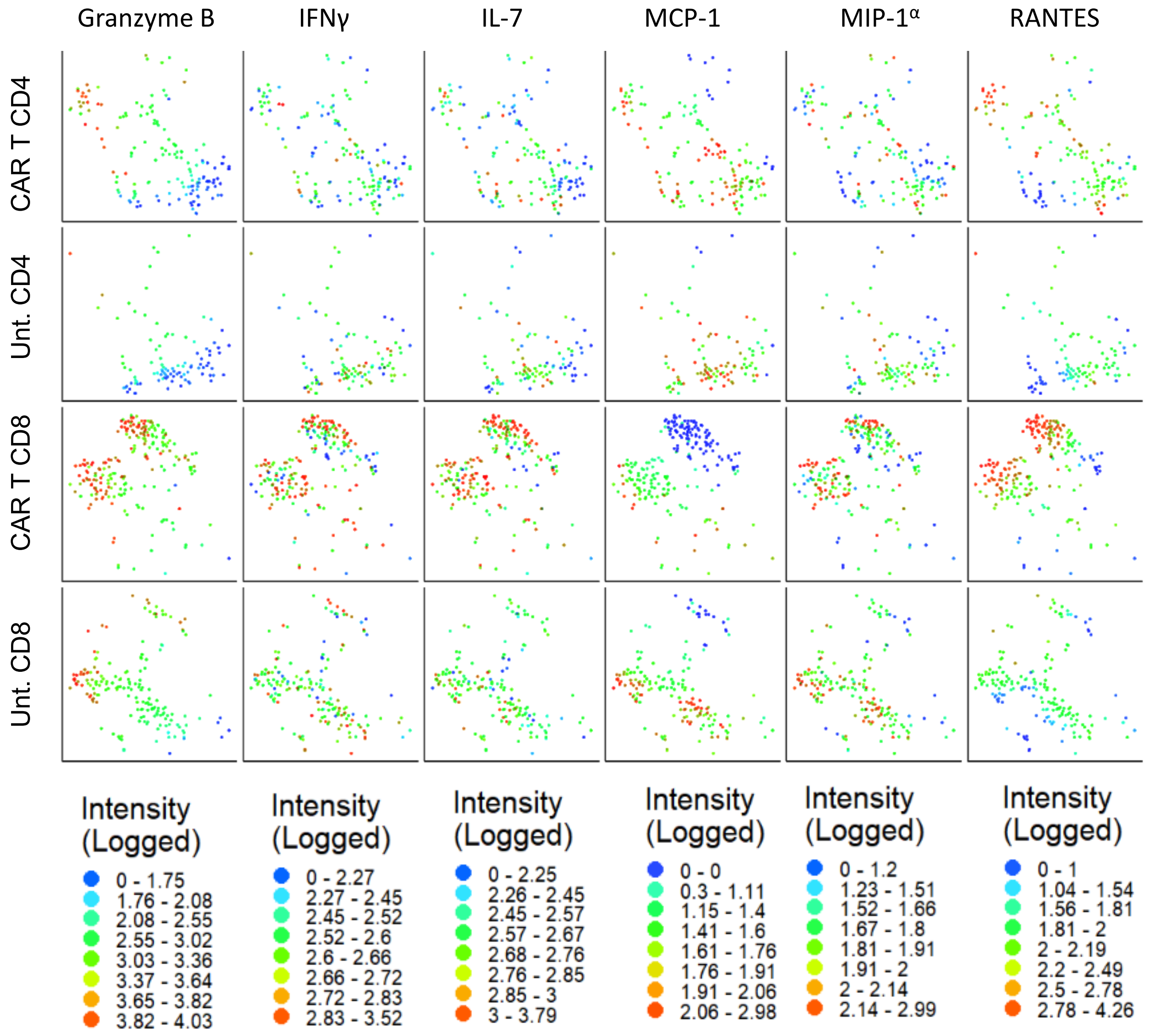

Supplementary Fig. S6: IsoPlexis single cell secretome data for untransduced and transduced CAR T-cells. Cells were sorted into CD4 and CD8 subpopulations and cocultured with GD3 synthase transfected HEK293 target cells before staining each T cell population. Cytokines detected among CD4 and CD8 untransduced and GD3 CAR T-cells are displayed for each detected cytokine, represented as tSNE plots. The units for the 2D tSNE represent the log transformed signal in RFU.

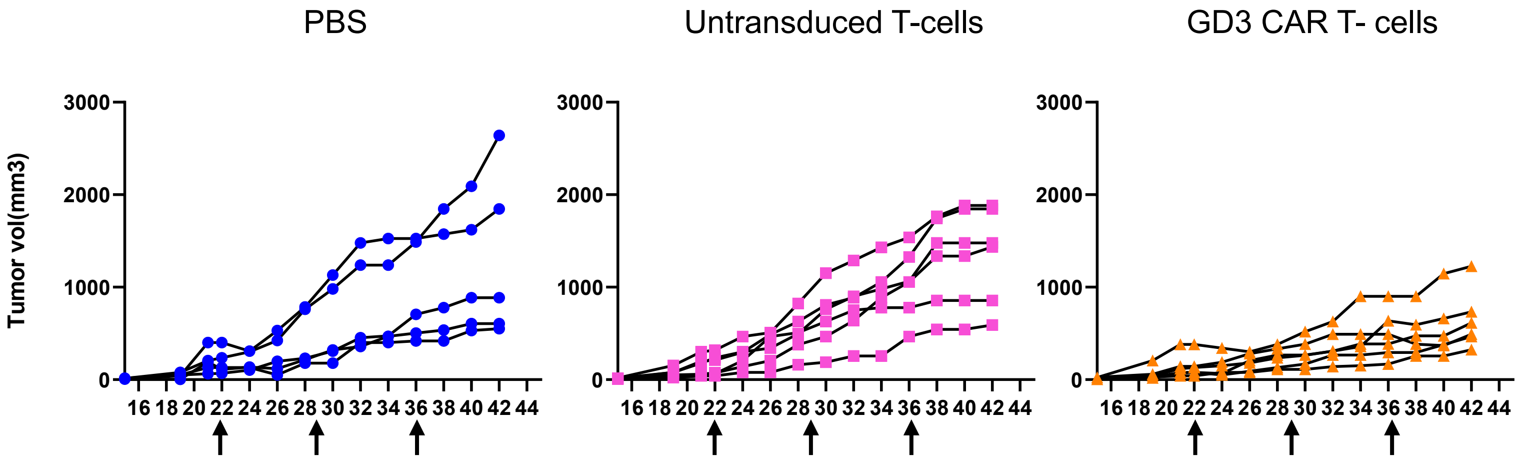

Supplementary Figure S7: Individual tumor measurements in immunodeficient mice. SCID/Bg mice challenged with 1 million LB-1 *Tsc2*$^{-/-}$ tumor cells and treated with vehicle or 1x10^6 untransduced T cells or GD3CAR-T cells (n=5). On days 22, 29, and 36 following tumor challenge (shown as days 1, 8 and 15 of treatment respectively in main figure 5B), mice were treated by adoptive transfer of T cells. Arrows indicate the T- cell injection time points.

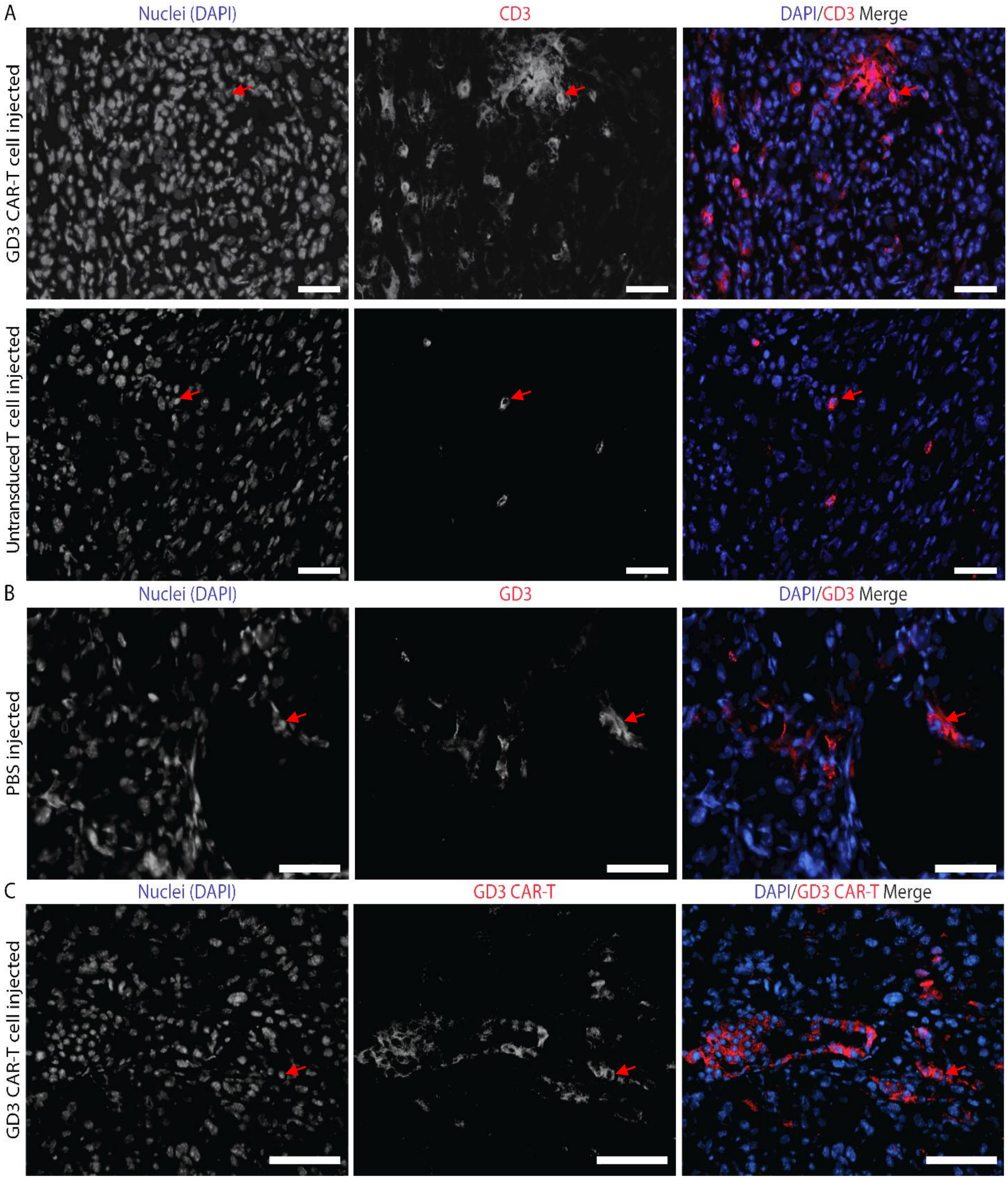

Supplementary Fig. S8: Immune cell infiltration in SCID-Bg tumors (A) CD3+ T cells infiltrating subcutaneous tumors after adoptive T cell transfer in SCID-Bg mice received untransduced or GD3 CAR-T cells. (B) Representative image of the GD3 expression in the subcutaneous *Tsc2-/-* tumor in a PBS-treated SCID-Bg mouse. (C) Representative image of the GD3 CAR-T cell detection in the CAR-T recipient SCID-Bg mice. Scale bar= 50 μm, n=4 or 5, red arrows indicate examples of stained cells.

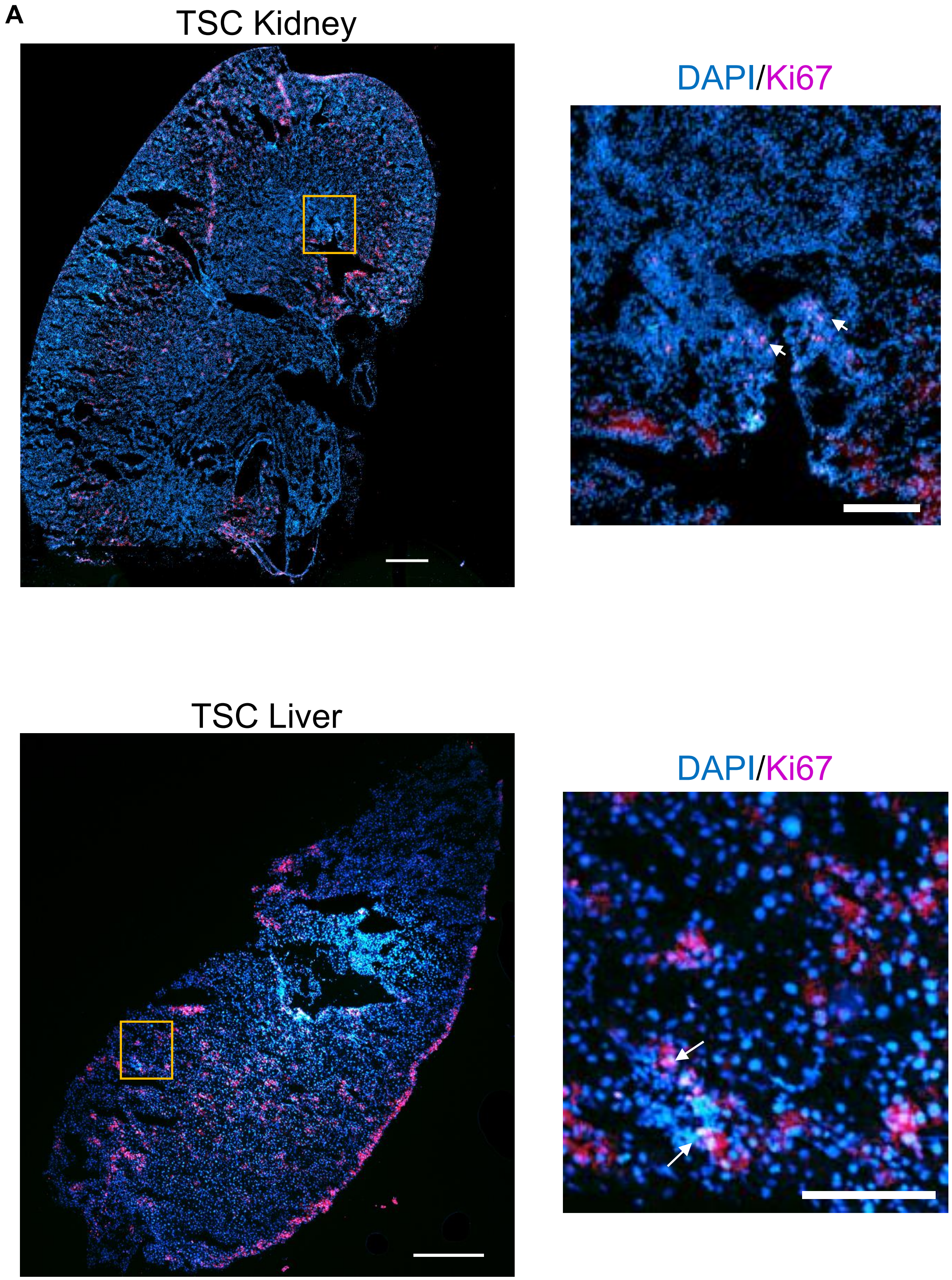

Supplementary Fig. S9: Cell proliferation TSC tumors in *Tsc2*$^{+/-}$ mice (A) Representative images of Ki67 cells staining in kidney and liver tissues of untransduced T cell injected *Tsc2*$^{+/-}$ mice. Scale bar= 100 μm in full tissue images, 25 μm in zoom inset images. Arrows indicate examples of stained cells.

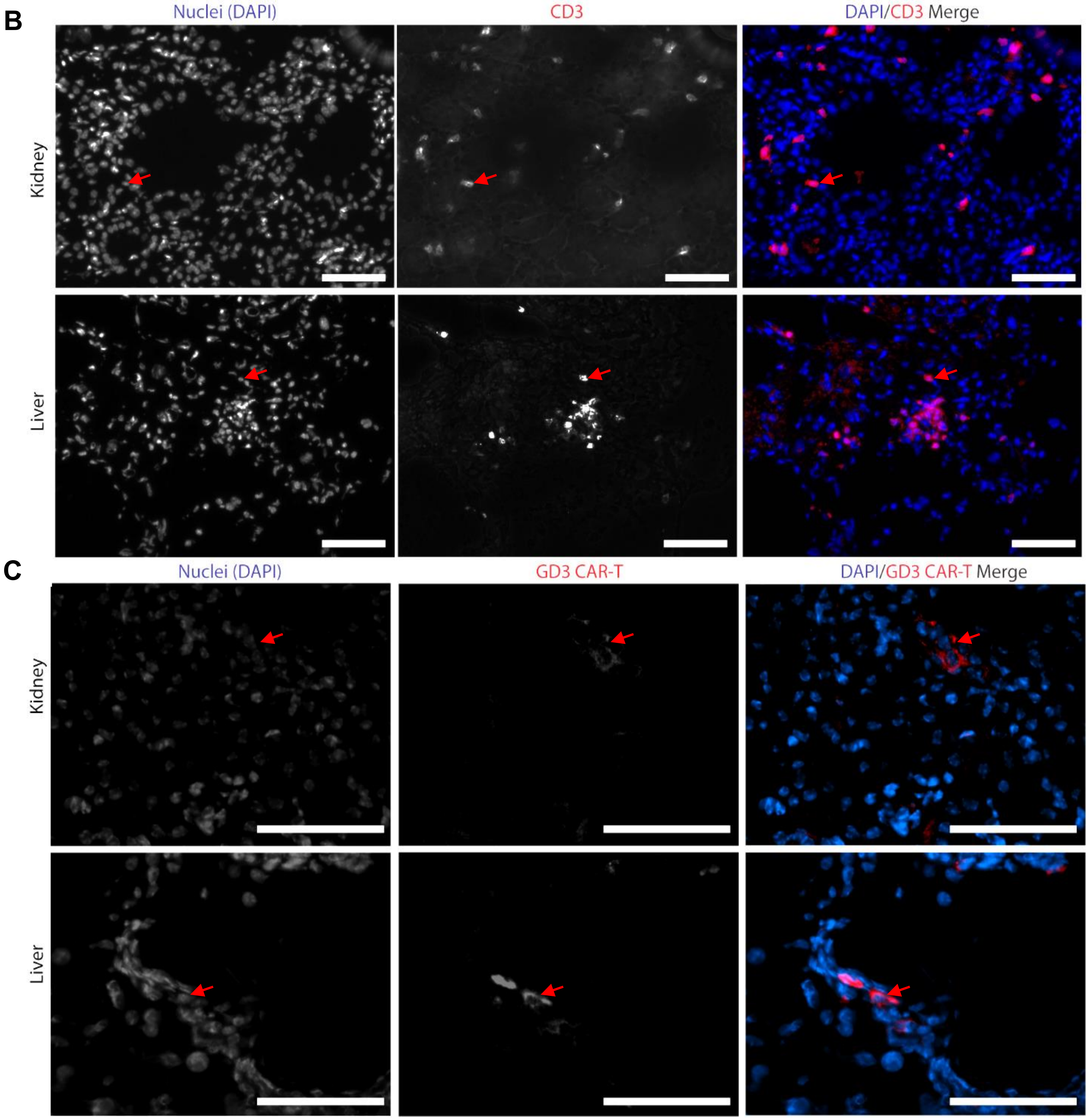

Supplementary Fig. S9: Immune cell infiltration in *Tsc2*$^{+/-}$ mice (B) Representative images of CD3 T cells staining in liver and kidney tissues of untransduced and GD3 CAR-T cell injected *Tsc2+/-* mice. (C) Representative images of GD3 CAR-T cells detection in liver and kidney tissues of the GD3 CAR-T cell injected *Tsc2+/-* mice. Scale bar= 50 µm, red arrows indicate examples of stained cells.

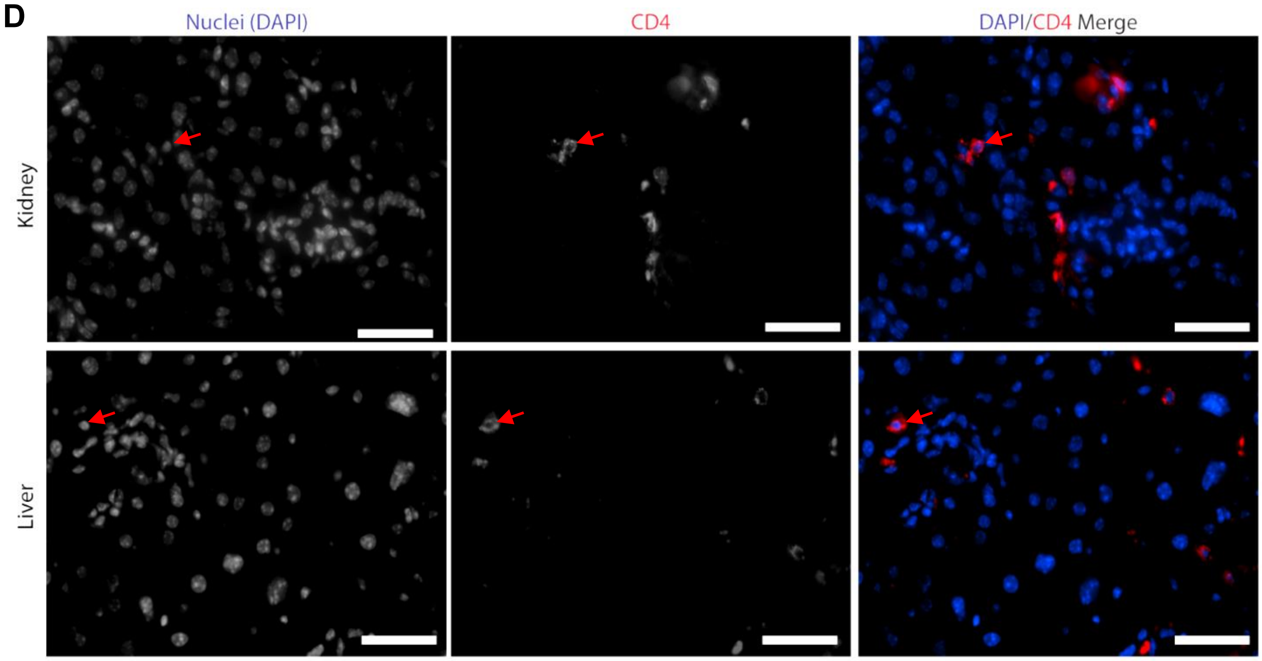

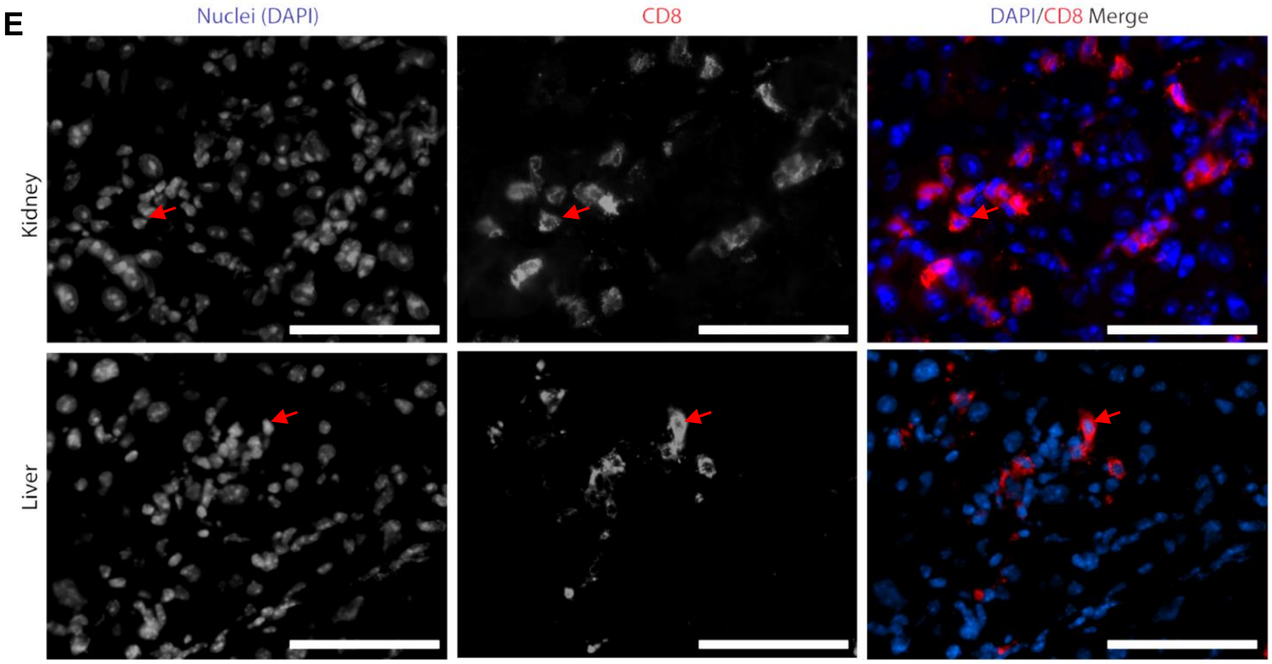

Supplementary Fig. S9, continued: Immune cell infiltration in *Tsc2*$^{+/-}$ mice. Representative images of CD4 (D) and CD8 T cells (E) staining in liver and kidney tissues of untransduced and GD3 CAR-T cell injected *Tsc2*$^{+/-}$ mice. Scale bar= 50 µm, red arrows indicate examples of stained cells.

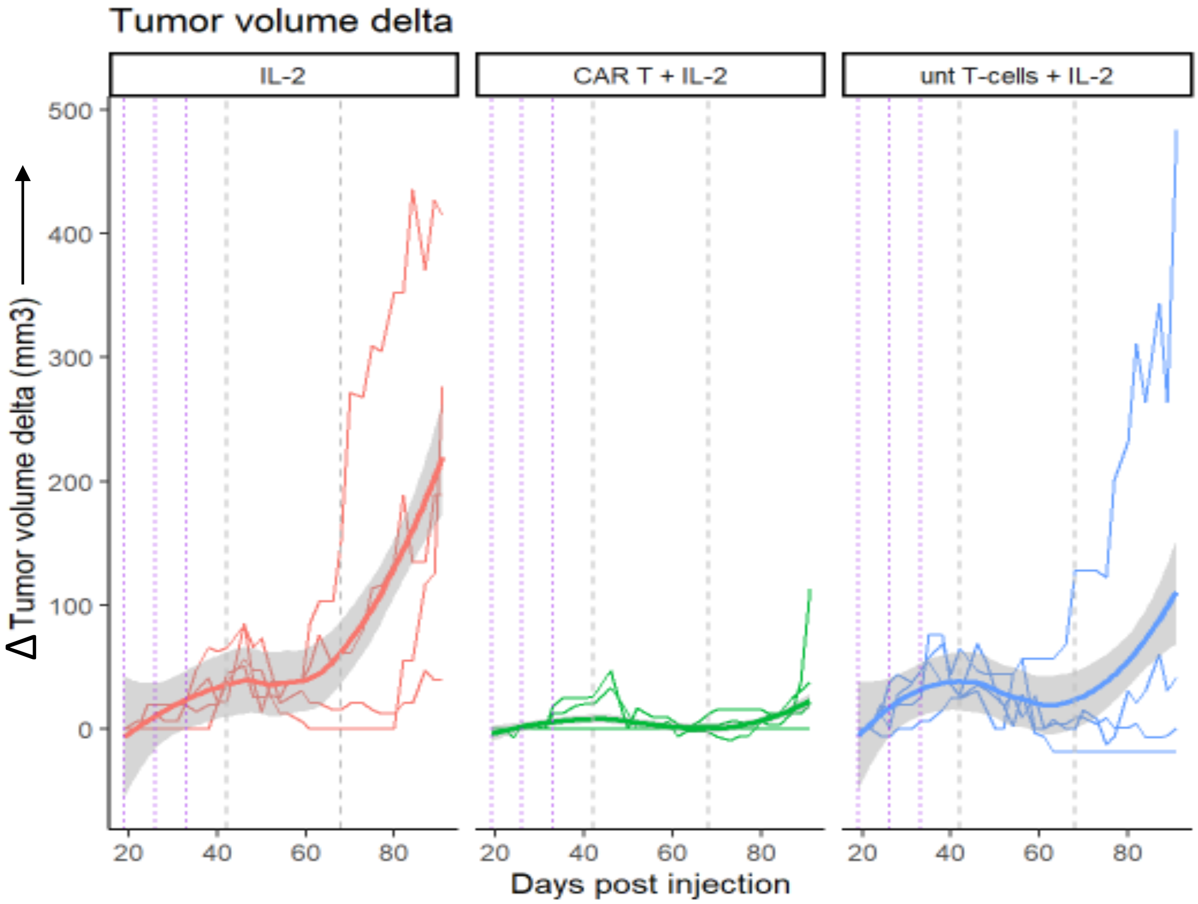

- Dotted purple line indicates treatment days
- Dotted grey line indicates knots of spline for modeling (at tertiles)

Supplemental Fig. S10: Progression free survival in tumor-challenged *Tsc2*$^{+/-}$ mice. Immunocompetent *Tsc2* $^{+/-}$ were subcutaneously challenged with 2.5x 10^6 *Tsc2*$^{-/-}$ (LB1) tumor cells. After 3 weeks, mice were lymphodepleted by 5 Gy irradiation and treated with 3 weekly injections of 10^6 T cells or PBS (blue line on X-axis). All mice received 30,000 IU of IL-2 IP 3 times per week. Tumor growth was followed for 10 weeks after treatment initiation. Compared to mice treated with IL-2 alone (AOC 4522, 95% confidence interval 2960-6084), the results indicate superior tumor control by CAR T- cells (AOC 361, 95% confidence interval 66-556) but not by untransduced T cells. (AOC 2601, 95% confidence interval 1293-3910). In a model of tumor growth with splines as shown in the figure, comparing tumor growth in the CAR T-cell group to that in the IL-2 control group, we see accelerated growth towards the end of the follow-up period with a p-value of 8.26 x10^{-5} (spline 4) and 1.21x10^{-6} (spline 5). The same holds true when comparing CAR T-cell treatment with the untransduced T cells group, with a p-value of 0.0244 in spline 5.

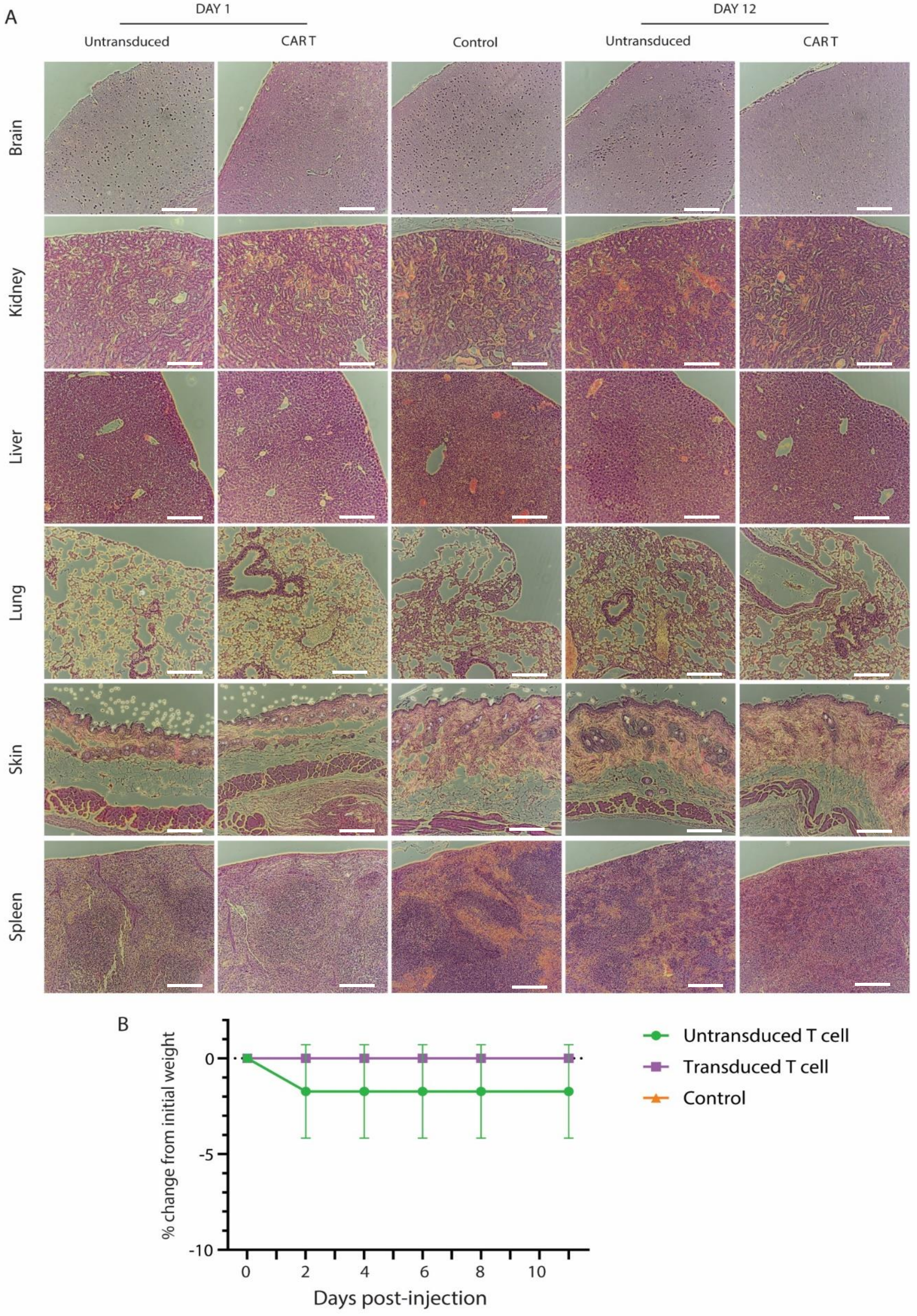

Supplementary Figure S11: No appreciable changes in tissue histology of animal weight after adoptive transfer. Ten months old mice were treated with or without 5x10^{6} untransduced or GD3CAR-T cells and were euthanized at different times and no changes in histology were found as shown in representative images. (A) Tissue histology up to 12 days post adoptive transfer from 3 mice each per time point was stained and analyzed on paraffin sections. A representative image from each time point is presented. (B) Weights for mice maintained over 12 days post transfer was were monitored over time without significant changes. Scale bar = 500um

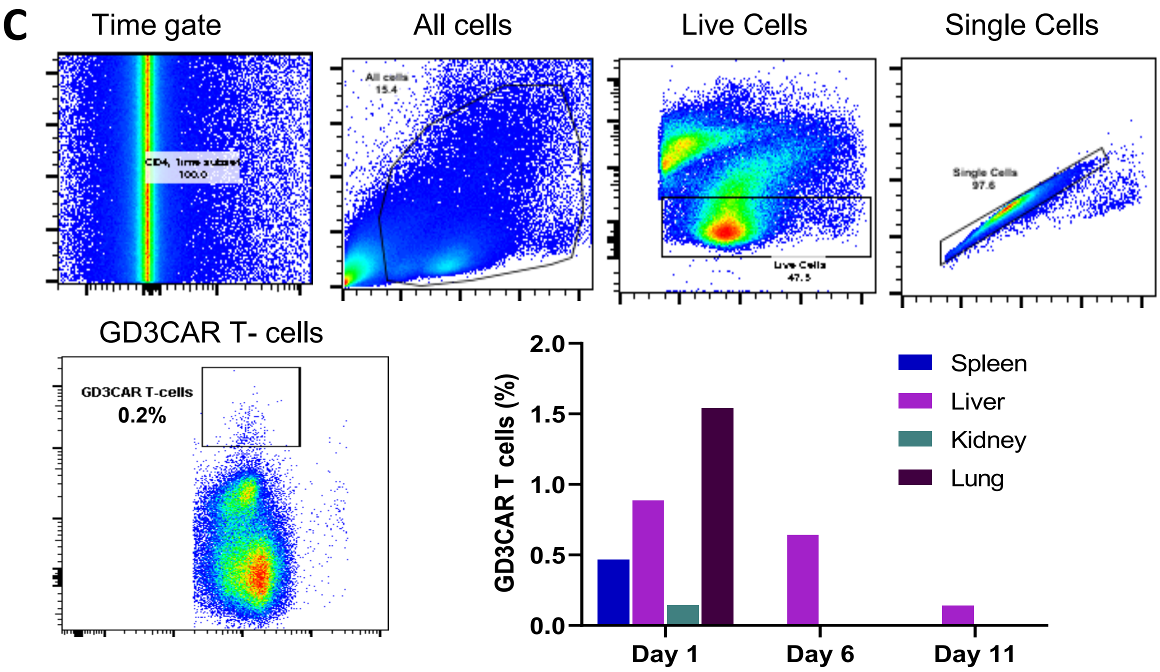

Supplementary Figure S11 continued: GD3CART-cell is safe for administration in *Tsc2*$^{+/-}$ mice. Ten months old *Tsc2*$^{+/-}$ mice were treated with 5 million GD3 CAR T-cells and were euthanized on day 1, 6 and 11 post T cell injection. (C) Detection of GD3 CAR T-cells in *Tsc2*$^{+/-}$ mice spleen 1 day post administration in FACS plots and percentage of GD3 CAR T-cells among total T-cells in spleen, liver kidney and lung of individual *Tsc2*$^{+/-}$ mice on days 1, 6 and 11 post T-cell administration. Pooled samples from 3 mice at each point was stained and analyzed and example FACS plots are presented.

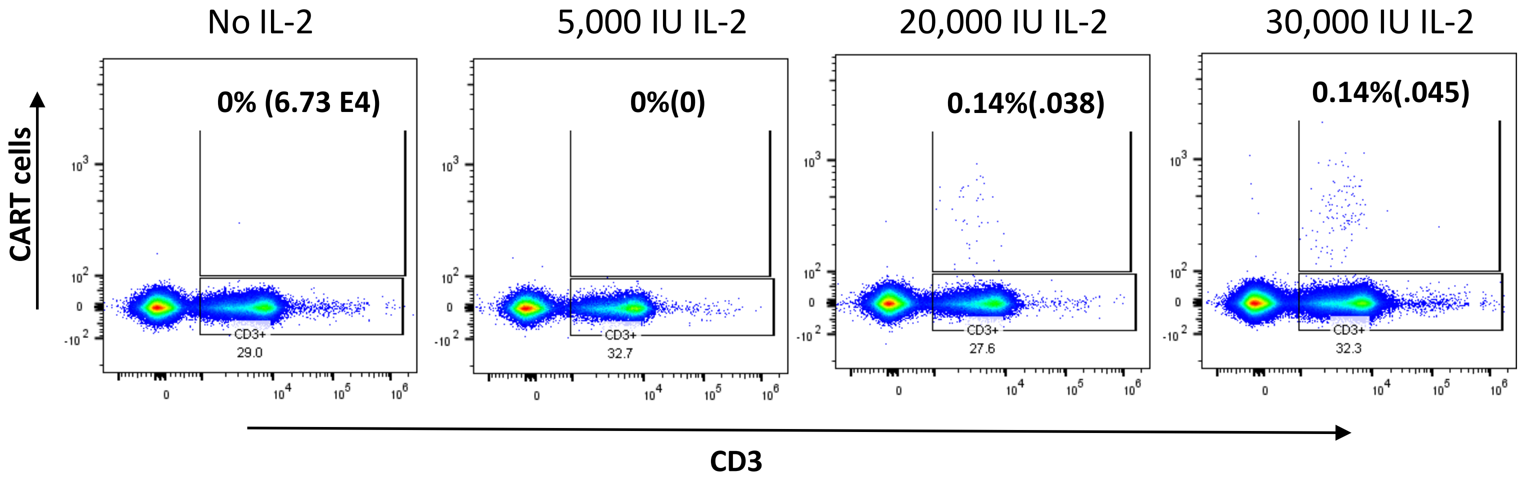

Supplementary Fig. S12: Persistence of GD3 CAR T-cells in vivo at different IL-2 dosages

Tsc2 $^{+/-}$ animals (10-11 weeks old) were lympho depleted with total body irradiation of 5Gy. After 24 hr, animals received 1 M GD3 CAR T-cells, pre stained with cell trace violet (CTV) and IL-2 at a dosage of 0, 5,000, 20,000 and 30,000 IU per animal. Animals were euthanized after 48 hr (n=3), splenocytes were collected and analyzed by FACS for CTV positive GD3 CAR T-cells as a percentage of the total T- cells and (between brackets) of total splenocytes. FACS plot from each IL-2 dosage is represented.
